# Novel loci and pathways significantly associated with longevity

**DOI:** 10.1038/srep21243

**Published:** 2016-02-25

**Authors:** Yi Zeng, Chao Nie, Junxia Min, Xiaomin Liu, Mengmeng Li, Huashuai Chen, Hanshi Xu, Mingbang Wang, Ting Ni, Yang Li, Han Yan, Jin-Pei Zhang, Chun Song, Li-Qing Chi, Han-Ming Wang, Jie Dong, Gu-Yan Zheng, Li Lin, Feng Qian, Yanwei Qi, Xiao Liu, Hongzhi Cao, Yinghao Wang, Lijuan Zhang, Zhaochun Li, Yufeng Zhou, Yan Wang, Jiehua Lu, Jianxin Li, Ming Qi, Lars Bolund, Anatoliy Yashin, Kenneth C. Land, Simon Gregory, Ze Yang, William Gottschalk, Wei Tao, Jian Wang, Jun Wang, Xun Xu, Harold Bae, Marianne Nygaard, Lene Christiansen, Kaare Christensen, Claudio Franceschi, Michael W. Lutz, Jun Gu, Qihua Tan, Thomas Perls, Paola Sebastiani, Joris Deelen, Eline Slagboom, Elizabeth Hauser, Huji Xu, Xiao-Li Tian, Huanming Yang, James W. Vaupel

**Affiliations:** 1 Center for the Study of Aging and Human Development and Geriatrics Division, Medical School of Duke University, Durham, North Carolina, USA; 2 Center for Healthy Aging and Development Studies, National School of Development, Peking University, Beijing, China; 3 BGI-Shenzhen, Shenzhen, China; 4 The First Affiliated Hospital, Institute of Translational Medicine, School of Medicine, Zhejiang University, Hangzhou, China; 5 Department of Rheumatology and Immunology, Shanghai Changzheng Hospital, The Second Military Medical University, Shanghai, China; 6 Business School of Xiangtan University, Xiangtan, China; 7 State Key Laboratory of Genetics Engineering & MOE Key Laboratory of Contemporary Anthropology, School of Life Sciences, Fudan University, Shanghai, China; 8 Department of Human Population Genetics, Institute of Molecular Medicine, Peking University, Beijing, China; 9 Department of Bio-Medical Engineering, School of Life Sciences, Anhui Medical University, Hefei, China; 10 Department of Sociology, Peking University, Beijing, China; 11 Department of Biomedicine, Aarhus University, Aarhus, Denmark; 12 Population Research Institute, Duke University, Durham, North Carolina, USA; 13 Duke Molecular Physiology Institute, Medical Center, Duke University, Durham, North Carolina, USA; 14 National Institutes of Geriatrics, Beijing Hospital, Ministry of Health, Beijing, China; 15 Department of Neurology, Medical Center, Duke University, Durham, North Carolina, USA; 16 School of Life Sciences, Peking University, Beijing, China; 17 James D. Watson Institute of Genome Sciences, Hangzhou, China; 18 Department of Biology, University of Copenhagen, Copenhagen, Denmark; 19 College of Public Health and Human Sciences, Oregon State University, USA; 20 The Danish Aging Research Centre, Unit of Epidemiology, Biostatistics and Biodemography, Institute of Public Health, University of Southern Denmark, Odense, Denmark; 21 Department of Experimental, Diagnostic and Specialty Medicine and Interdepartmental Centre ‘L. Galvani’, University of Bologna, Bologna, Italy; 22 Geriatrics Section, Department of Medicine, Boston University School of Medicine, Boston, MA, USA; 23 Department of Biostatistics, Boston University School of Public Health, Boston, MA, USA; 24 Department of Molecular Epidemiology and Netherlands Consortium for Healthy Ageing, Leiden University Medical Center, Leiden, The Netherlands; 25 Max Planck Institute for Demographic Research, Rostock, Germany

## Abstract

Only two genome-wide significant loci associated with longevity have been identified so far, probably because of insufficient sample sizes of centenarians, whose genomes may harbor genetic variants associated with health and longevity. Here we report a genome-wide association study (GWAS) of Han Chinese with a sample size 2.7 times the largest previously published GWAS on centenarians. We identified 11 independent loci associated with longevity replicated in Southern-Northern regions of China, including two novel loci (rs2069837-*IL6*; rs2440012-*ANKRD20A9P*) with genome-wide significance and the rest with suggestive significance (*P* < 3.65 × 10^−5^). Eight independent SNPs overlapped across Han Chinese, European and U.S. populations, and *APOE* and *5q33.3* were replicated as longevity loci. Integrated analysis indicates four pathways (starch, sucrose and xenobiotic metabolism; immune response and inflammation; MAPK; calcium signaling) highly associated with longevity (*P* ≤ 0.006) in Han Chinese. The association with longevity of three of these four pathways (MAPK; immunity; calcium signaling) is supported by findings in other human cohorts. Our novel finding on the association of starch, sucrose and xenobiotic metabolism pathway with longevity is consistent with the previous results from Drosophilia. This study suggests protective mechanisms including immunity and nutrient metabolism and their interactions with environmental stress play key roles in human longevity.

Human longevity is a complex trait determined by both genetic and environmental factors, and the genetic influence increases with the higher ages^1^,^2^,^3^,^4^. Prior studies showed that most centenarians compress disability period towards the ends of their lives, suggesting that they avoid diseases of aging and associated disability until old ages^5^,^6^,^7^. Furthermore, the offspring of centenarians have significantly better health compared to peers^8^,^9^. Hence, centenarian genomes may harbor genetic variants associated with health and longevity^10^. In recent years, a number of genome-wide association studies (GWAS) in North America and Europe have been conducted on longevity, but only two loci have been identified associated with longevity at a genome-wide significant level: the well-known *TOMM40/APOE/APOC1* locus which is negatively associated with longevity^4^,^10^,^11^, and a second locus on chromosome *5q33.3*, which was identified in a recent GWAS by Deelen *et al*.^10^.

While interest in this topic is strong, the field is hindered by a lack of databases with both genotypic and phenotypic information and sufficiently large sample sizes of centenarians^4^,^11^. The largest sample size of centenarians in GWAS studies published to date was 801, in which the study found only one genome-wide significant single nucleotide polymorphism (SNP)^4^. Much larger samples of centenarians and ethnically matched controls are needed for genome wide significant discoveries of genetic associations with longevity^3^,^11^.

To expand the catalogue of longevity-associated loci and gain a better understanding of the influences of genes and biological pathways on longevity, we performed a GWAS using the samples derived from the Chinese Longitudinal Healthy Longevity Survey (CLHLS) (section M1 of Methods). The present study involved 2,178 Han Chinese centenarians, which is about 2.7 times the largest sample size of ever-published GWAS on centenarians^4^, and 2,299 middle-age controls after sample filtering, implying reasonably good power in our study (section M2 of Methods).

Our work also represents the first GWAS of longevity in Asian populations and in developing countries more generally. There were about five centenarians per million in China in the 1990s, compared with 50 per million in Western Europe in the same period^12^. Han Chinese centenarians may be more likely to have longevity-associated and/or disease-preventive genes than their centenarian counterparts in the West since they survived the brutal mortality regimes of the past when famine, wars, and starvation operated on birth cohorts of many millions. Unlike Western countries that received many international immigrants from other parts of the world resulting in relatively heterogeneous genetic compositions even within ethnic groups, China received very few international immigrants. Consequently, even though one might expect population substructures because of the long history of interaction with surrounding minority Chinese ethnic groups^13^, Han Chinese are relatively more homogenous in genetic composition compared to their Western counterparts. For example, it has been estimated that the average of genetic differences measured by F-statistics (F_ST_)^14^ between Han Chinese population samples (F_ST_ = 0.002) is much lower than that among European populations (F_ST_ = 0.009)^13^. Thus, GWAS with large samples of Han Chinese centenarians and middle-age controls are expected to be instrumental for identifying genetic variants related to longevity.

## Results

### Population characteristics and analytical strategy

Standard quality controls are detailed in section M2 of Methods. Based on existing literature on Chinese genetic studies^15^ and our principal component analysis (PCA) (section M4 of Methods, Supplementary Figs 1 and 2), we stratified the samples into two independent GWAS datasets of Southern and Northern regions, with 1,063/887 centenarians/controls in the Southern dataset, and 1,115/1,412 centenarians/controls in the Northern dataset (Supplementary Table 1). The genomic inflation factors (λ) in the Southern, Northern and combined datasets were 1.022, 1.010 and 1.022, respectively, indicating that the effects of population stratification on genetic analysis are well controlled (Supplementary Fig. 3)^16^. To minimize both false-positive and false-negative rates, we applied a novel bi-directional discovery-evaluation strategy^17^, which fully uses the two independent GWAS datasets of Southern and Northern regions by parallel analysis: one as the discovery dataset and the other as the evaluation dataset, and vice versa. As described in the section M5 of Methods, when analyzing two independent GWAS datasets, the classic uni-directional discovery-replication approach of assigning one GWAS dataset as discovery and another GWAS dataset as replication would result in a higher false-negative rate missing a substantial number of replicated SNPs that have a p-value higher than the threshold and lower than the nominal significance level in the discovery GWAS dataset but that reach the threshold significance level in the replication GWAS dataset. Our bi-directional discovery-evaluation strategy enables us to avoid the higher false-negative rate.

**Figure 1:**
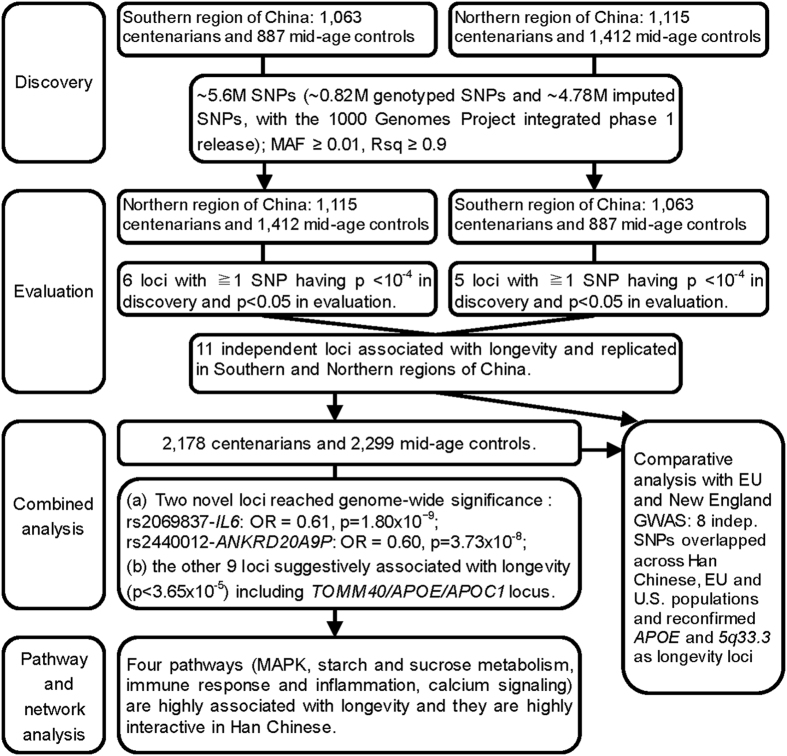
A flow chart of the consecutive analysis steps.

We defined *a priori* discovery threshold of *P* < 10^−4^ as the discovery threshold p-value based on recent relevant literature as cited and discussed in section M5 of Methods. We used a nominal significance of *P* < 0.05 in the evaluation stage. We performed GWAS using logistic regression implemented in PLINK (version 1.06) and adjusted for the top two eigenvectors and sex (section M6 of Methods). Using the SNPs identified in the bi-directional discovery-evaluation analyses, we performed a combined analysis comparing Han Chinese 2,178 centenarians with 2,299 mid-age controls, adjusted for the geographic stratification of Southern and Northern regions in addition to sex. In addition, we performed a meta-analysis, treating the two independent datasets from Southern and Northern regions of China as two groups. We then conducted an evaluation/comparison analysis of our results with the data from longevity GWAS of European Union (EU) longevity genetics consortium and the U.S. New England centenarians study (NECS). To extend the foregoing SNP association analyses towards understanding of biological processes underlying longevity, we conducted pathway and network analyses. A flow chart of the consecutive analysis steps is depicted in Fig. 1.

### Association analyses of SNPs with longevity in Han Chinese

**Figure 2:**
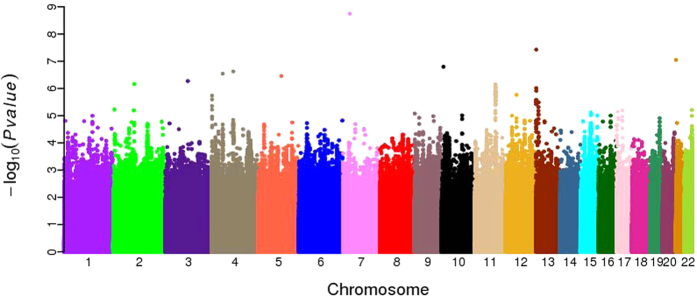
Manhattan plot showing the results of the association with longevity in the combined GWAS dataset.

In the bi-directional discovery-evaluation analysis, we identified 11 independent loci (Table 1 and Fig. 2) that were replicated in the independent GWAS datasets of Southern and Northern regions of China. All 11 loci were associated with longevity with p < 3.65 × 10^−5^ in the combined dataset, of which two loci, rs2069837 (chromosome 7p15.3, *IL6*, *P* = 1.80 × 10^−9^) and rs2440012 (chromosome 13q12.12, *ANKRD20A9P*, *P* = 3.73 × 10^−8^) reached genome-wide significance (*P* < 5 × 10^−8^) (Table 1 and Fig. 2). *IL6* has been linked to longevity previously^18^,^19^, but rs2069837 identified in this study is a novel signal within the *IL6* locus. The minor allele of rs2069837 is significantly less frequent among centenarians than middle-age individuals in Han Chinese (odds ratio = 0.61; *P* = 1.80 × 10^−9^), suggesting the effect of this locus on longevity is deleterious. This outcome is consistent with published findings that the *IL6* gene functions as an inflammatory biomarker of functional decline and poor health outcomes including increased mortality risk^20^,^21^. The other novel SNP rs2440012 is located in *ANKRD20A9P*, a pseudogene that is affiliated with the long non-coding RNAs (lncRNA) class. The biological function of this particular non-coding transcript variant remains to be characterized.

Among the other 9 replicated loci associated with longevity at a suggestive significant level (*P* < 3.65 × 10^−5^), the *TOMM40/APOE/APOC1* locus is particularly interesting since its relationship with longevity is well known^4^,^10^,^11^, and we discuss it in more detail below. The remaining 8 novel replicated loci include MIR3156-3 (rs145672791, 21q11.2, 28 kb downstream), *AKR1C2* (rs61856137, 10p15.1, 27 kb upstream), *FAM13A* (rs2704588, 4q22.1, intronic), *BEND4* (rs1487614, 4p13, 114 kb upstream), *EPHA6* (rs10934524, 3q11.2, 383 kb upstream), *ASIC2* (rs11658235 and rs7212444, 17q12, intronic) and *OLFM4* (rs9568833, 13q14.3, 200 kb downstream).

The meta-analysis results (last column in Table 1) are in full agreement with those of the combined analysis adjusted by a binary covariate of Southern and Northern regions, except the *P* values are slightly higher. Together, the 11 loci associated with longevity explained 3.38% of the variance in surviving to ages 100 + from middle-age, with each locus contributing from 0.39% (rs9568833-*OLFM4*) to 1.0% (rs2069837-*IL6*) of the variance based on effect estimates in the combined analysis, using the restricted maximum likelihood (REML) method^22^.

Notably, gender-specific association analysis for the 11 SNPs listed in Table 1 showed the same direction of effect in men and women with mostly very similar odds ratios (Supplementary Table 2). All of the 11 SNPs had a p-value of p < 10^−3^ in women. Ten of the 11 SNPs achieved nominal significance (*P* < 0.05) in men and one SNP had a *P* = 0.0549 in men (Supplementary Table 1). The cross-gender similarities in effect direction/size on longevity of these 11 SNPs, which are replicated between discovery and evaluation datasets, is consistent with the results of the other GWAS on longevity^10^,^4^, and the gender differences of the not-replicated SNPs is out of scope of this article and will be investigated in our subsequent study.

### Comparison of current results with longevity GWAS studies of different ethnicities

To investigate the similarities and differences of genetic associations with longevity across the Han Chinese, European and U.S. ethnicities, we performed an evaluation/comparison analysis by using our Han Chinese GWAS as discovery and two European/American studies as evaluation. The two GWAS studies used for evaluation were the EU longevity genetics consortium, with a population of 5,406 aged 90+ and 15,112 controls aged <65 that included 14 studies from the Netherlands, Denmark, Iceland, Germany, Italy, the United Kingdom and Sweden^10^, and the U.S. New England centenarian study (NECS) of 1,030 long-lived individuals (age 95 and older for males and 100 and older for females) and 368 controls with a mean age of 79.9^4^.

The results presented in Supplementary Table 3 show that among the 11 SNPs associated with longevity and replicated in Southern and Northern GWAS datasets of CLHLS, six SNPs had available data in at least one of the two other longevity GWAS studies, and one SNP is not available in the other two longevity GWAS but it has a proxy SNP (r^2^ = 0.97) from the European population SNPs database (see Table 1 and its note (3)). The SNP rs405509 in *APOE* replicated in Southern and Northern GWAS datasets of CLHLS was also replicated in both EU (*P* = 2.75 × 10^−06^) and New England GWAS (*P* = 2.46 × 10^−03^), identifying a novel SNP-specific replication (Supplementary Table 3).

We conducted additional evaluation/comparison analysis of the other 723 SNPs associated with longevity at a suggestive significance level (*P* < 10^−4^) in the Han Chinese GWAS combined dataset. Among the 723 SNPs, 267 were available in the EU GWAS, and 37 were available in the New England GWAS. Of these, eight independent SNPs associated with longevity (*P* < 10^−4^) in the Han Chinese GWAS overlapped with at least nominal significance (*P* < 0.05) in at least one of the longevity GWAS in Europe (8 SNPs) and New England (2 SNP). Among these overlapped SNPs are rs405509 in *APOE* described above and rs4420638 in the *TOMM40/APOE/APOC1* locus, which was replicated across continents with strong supporting evidence in all of the three GWAS datasets: *P* = 7.85 × 10^−5^ in the Han Chinese, *P* = 4.09 × 10^−21^ in the EU and *P* = 1.03 × 10^−09^ in the New England.

In addition, we evaluated 54 SNPs associated with longevity (*P* < 10^−4^) discovered in the four previously published major studies of GWAS on longevity^4^,^10^,^11^,^23^ and found that 44 of them were available in Han Chinese GWAS. Among these 44 SNPs, thirty-nine were not associated with longevity in the Han Chinese (p > 0.05, data not shown but available upon request), and four SNPs in the *TOMM40/APOE/APOC1* region reported in EU and New England GWAS replicated in the Han Chinese GWAS with *P* < x 10^−4^ (Supplementary Table 5). Furthermore, our Han Chinese GWAS identified an additional 10 SNPs with *P* < 10^−4^ in the *TOMM40/APOE/APOC1* region; and among them, 8 SNPs were in very high linkage disequilibrium (r^2^ = 1.0 or 0.99) with the significant SNPs reported in the EU and New England GWAS on longevity, and an additional 2 SNPs have not been reported before (Supplementary Table 5). Given the large number of associated SNPs in the *TOMM40/APOE/APOC1* region we performed a conditional analysis to identify independent association signals at this locus. The top independent association was rs405509 with *P* = 3.64 × 10^−5^. When conditioning on rs405509, we observed a secondary independent association at rs71352238 (*P*_conditional_ = 2.1 × 10^−4^) (Supplementary Fig. 4). After adjusting for rs405509 and rs71352238, we observed no other significant associations (*P* > 0.01) at this locus (Supplementary Fig. 4). These results demonstrate that the two independent associations in the *TOMM40/APOE/APOC1* account for all of the remaining associated signals in this region in the CLHLS GWAS.

The genome-wide significant longevity locus *5q33.3* (rs2149954, *P* = 1.74 × 10^−8^) reported in the EU GWAS^10^ showed the same direction of effect with *P* = 0.02 in the Han Chinese GWAS. We also found 4 SNPs of the reported longevity gene *FOXO3* are associated with longevity with a *P* < 0.04 and odds ratio around 1.2 in Han Chinese (data not shown, but available upon request).

The genome-wide significant locus identified in our Han Chinese GWAS, rs2069837 (*P* = 1.80 × 10^−9^), is located in the intronic region of the *IL6* gene on chromosome 7p15.3 (Table 1). The SNP rs2069827 in *IL6* that was reported previously as associated with longevity^18^,^19^ was not found in Han Chinese, but it is rather common in European populations based on 1000genome annotation in HaploReg V2. Similarly, our identified SNP rs2069837 has a MAF of 0.14 in Asian populations but 0.09 in the European population, indicating that the previously reported SNP rs2069827 is a European-specific longevity associated genotype and our identified SNP rs2069837 could be a Han Chinese-specific longevity associated genotype. The finding that these two genetic variants of *IL6* contribute to longevity in opposite directions in Han Chinese versus Europeans emphasizes the impact of genetic heterogeneity on longevity across ethnicities.

In brief, our comparative analysis indicates both considerable similarities and differences in genetic associations with longevity between the Chinese, European and U.S. populations. Further cross-national meta-analysis is warranted to develop an in-depth understanding of the cross-ethnics genetic associations with human longevity.

### Pathway and network analyses

We conducted pathway analyses by applying i-GSEA4GWAS, an improved gene set enrichment analysis (GSEA) for GWAS^24^ (section M7 of Methods). Twenty-five canonical pathways were ranked as significantly enriched (FDR < 0.05 and corrected p-value ≤ 0.004) and associated with longevity (Supplementary Table 6). These can be functionally classified into 4 major pathways: starch, sucrose and xenobiotic metabolism (10 enriched pathways), immune response and inflammation (7 enriched pathways), MAPK (4 enriched pathways) and calcium signaling (2 enriched pathways), plus 2 other significant enriched pathways (see Supplement-Table 6). Our finding of the four major significant pathways for longevity is generally similar to the results from Alzheimer’s disease GWAS studies, in which over 20 genes with statistically-significant association signals^25^ were mapped to relatively few major significant pathways including lipid metabolism, inflammatory response, endocytosis and immune response^26^.

Among the four major pathways identified in our present study, p38 MAPK^27^ and immunity^28^ were linked to longevity previously. In particular, p38 MAPK acts downstream of the IL-6 receptor, and that may account for this association. p38 MAPK also acts upstream of FOXO, which, as discussed above, has been associated with longevity in previous GWAS. Moreover, we also note that p38 MAPK can be activated by AMPK, which is upstream of mTOR, and activation of mTOR has been shown to extend life span in C. elegans^29^, mice^30^ and yeast^31^. In common with our results, mTOR signaling was identified as significant for the ageing phenotype by a recent study^32^.

Before the current study, starch, sucrose and xenobiotic metabolism and calcium signaling had not been identified as being associated with human longevity in the European and American GWAS. The Chinese diet is high in carbohydrates, mainly starch and sucrose, and is usually low in fat. Sucrose is a heterodimer of glucose and fructose, while starch is a glucose polymer, and both molecules are degraded in the gut. The liberated glucose generates an insulin response. Fructose does not generate an insulin response but when it is consumed in excess it contributes to multiple chronic metabolic diseases. Fructose metabolism also can promote reactive oxygen species (ROS) formation, which leads to cellular dysfunction and aging^33^. Thus, our novel finding that the starch, sucrose and xenobiotic metabolism pathway is significantly associated with human longevity in Han Chinese is interpretable, because the favorable defensive genotypes carried out by the Chinese centenarians may interact with their high-carbohydrates diet to achieve extreme longevity. Furthermore, this novel finding is consistent with the hypothesis that dietary sources of carbohydrates, mainly sucrose and starch, may affect life span in Drosophilia^34^.

Altered calcium homeostasis contributes to neurodegenerative diseases of aging, including Alzheimer’s disease (AD), and pharmacological inhibitors of calcium signaling have been shown to rescue structural plasticity defects in AD murine diseased neurons^35^. Our analysis suggests that calcium signaling has a potentially important impact on prolonging longevity.

Noteworthy, support for the relevance to longevity of three of the four major significant pathways found in our Han Chinese GWAS (MAPK, immunity and calcium signaling) has been most recently provided by Tan *et al*.^36^ as significant pathways in their longitudinal epigenome-wide association study (EWAS) of Danish twins with a mean age of 76 years at entry and, followed for 10 years. Interestingly, gene expression levels for the four significant pathways found in the Han Chinese GWAS were reported as differentially regulated during aging in C. elegans^37^. These consistent findings of the important pathways for both genetic association with longevity as well as differential functional regulation over the aging process in both humans and animal models suggest that the functional coordination of these 4 major pathways have a profound impact on longevity and aging.

**Figure 3:**
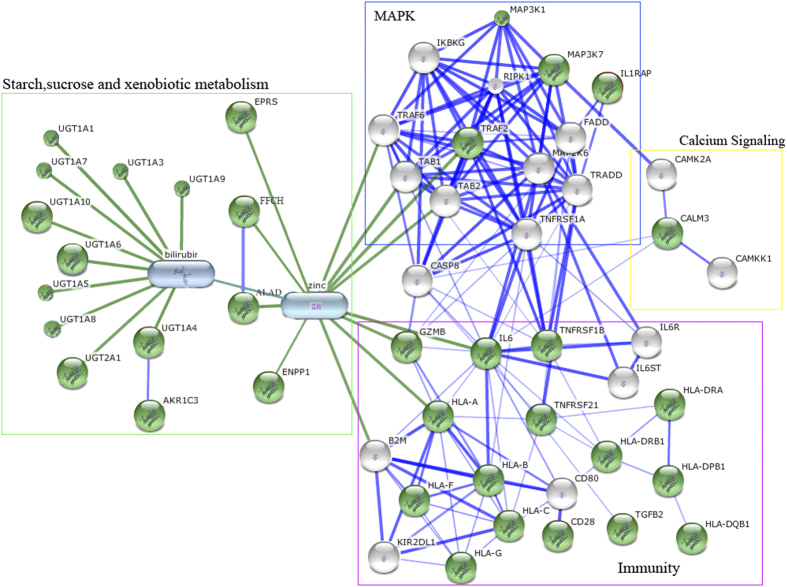
Gene and Pathway networks in longevity traits. Stronger associations are represented by thicker lines. Protein-protein interactions are shown in blue, protein-chemical interactions and chemical-chemical interactions in green. Note: The green nodes mark 35 highly-represent genes in the four main pathways, the white nodes mark highly interacted genes with these 35 genes. The four main pathways are shown with colored rectangles.

To further understand the interconnection of genes in the identified major pathways, we performed network analysis in STITCH^38^ (section M8 of Methods) and identified 35 genes (Supplementary Table 7) that are highly represented across the 25 enriched pathways. These 35 highly-represented genes include gene family members *UGT1A* (the gene cluster that defends against organic molecules, such as small molecule toxins) and *HLA* (the gene functions in immunity protecting against pathogens), which indicate potential defensive mechanisms that are important for longevity. As shown in the connectivity map (Fig. 3), we find that the four major pathways that mediate defensive mechanisms and have a role in longevity, are highly interconnected. For example, *IL6*, a top ranked gene in both our SNPs and pathway analyses, is a key gene in immune response and inflammation and activating p38 via IL6 receptor. The p38 MAPK pathway, in turn, mediates pathogen-specific responses by regulating expression of many protective genes related to immune response, contributing to longevity^39^. Involvement of the both of *UGT1A* gene cluster, which detoxifies small molecules, and the HLA family, lend further support to the notion that defensive mechanisms are critical for successful longevity.

Taken together, our data strongly suggest that the longevity trait represents a complex interplay of multiple genes and pathways interacting with the environment, notably diet, that converge on specific biological processes.

### Analysis of regulation of gene expression by associated variants

We examined the potential effects of the 11 SNPs or their proxies (r^2^ ≧ 0.6) in our study on gene expression using several eQTL databases (section M9 of Methods). Two SNPs in moderate LD (r^2^ = 0.61) with rs11658235 (*ASIC2*), i.e. rs7224279 and rs11658301, showed significant trans-eQTL associations (*P* = 3.88 × 10^−9^) in human brain cerebellum tissue samples (Supplementary Table 9), but we did not find statistically significant eQTLs for other longevity-associated variants. The findings of our SNP analysis and this eQTL analysis suggest *ASIC2* locus in brain cerebellum plays a role in mediating longevity phenotype.

We also investigated whether the SNPs with a significance level of *P* < 10^−3^ in the 35 genes highly represented across pathways were associated with gene expression using eQTL data. By querying three publically available databases (GTEx, seeQTL, Chicago eQTL), we mapped multiple SNPs with highly significant cis-eQTLs in human tissue samples to three genes, including *MAP3K1, HLA-B* and *HLA-DPB1* (supplementary Tables 10–12).

### Overlap between association signals and regulators of transcription

By querying the RegulomeDB database^40^, which includes high-throughput, experimental datasets from ENCODE (Encyclopedia of DNA Elements data), we found rs405509 upstream of *APOE* exhibited a strong cis-effect on ZNF226 expression in lymphoblastoid cell lines^41^. Three SNPs in two other genes also mapped to regulatory regions, including rs2069837 in *IL6*, and rs7212444 and rs8064775 in *ASIC2*. These variants showed evidence of transcription factor binding and DNase I hypersensitivity peaks.

## Discussion

Here, we summarize and discuss our key findings. First, our GWAS of Han Chinese identified 11 independent loci associated with longevity and replicated in two independent GWAS datasets from Southern and Northern regions of China (Table 1). More specifically, we identified two novel loci (rs2069837-*IL6* and rs2440012-*ANKRD20A9P*) that reached genome-wide significance, plus 9 suggestively significant loci (*P* < 3.64 × 10^−5^), including rs405509 in *APOE*, which is a well-known locus negatively associated with longevity^42^. Our strongest novel signal is for the rs2069837 SNP (*P* = 1.80 × 10^−9^) within the *IL6* locus at 7p15.3, negatively associated with longevity in the Han Chinese, which is consistent with previous findings that the *IL6* gene functions as an inflammatory biomarker of poor health outcomes^20^,^21^. An earlier Dutch prospective cohort study of longevity reported a different SNP in *IL6*, rs2069827, which was positively associated with longevity^18^. The analyses showed that the previously reported SNP rs2069827 is European-specific and our newly identified SNP rs2069837 could be a Han-Chinese-specific longevity associated genotype. Another strong novel locus that reached genome-wide significance in Han Chinese GWAS was *ANKRD20A9P* (ankyrin repeat domain 20 family, member A9), a pseudogene which is affiliated with the lncRNA class. The biological function of this particular variant remains to be characterized.

Secondly, we identified 8 independent SNPs associated with longevity with *P* < 10^−4^ in Han Chinese GWAS that overlapped results from the European and/or New England longevity GWAS, with at least nominal significance (*P* < 0.05). We also confirmed 4 previously reported longevity-associated SNPs and identified 2 independent associations with longevity in the well-known *TOMM40/APOE/APOC1* LD region. Our findings provided additional new signals and replicated the negative association of the *TOMM40/APOE/APOC1* LD region with exceptional longevity in Han Chinese, and confirmed *5q33.3* as a longevity locus that was previously identified in EU-GWAS. In short, we demonstrated both similarities and differentials in genetic associations with longevity across continents and ethnicities.

Thirdly, at the pathway level, our study identified 4 major pathways that influence longevity, including starch, sucrose and xenobiotic metabolism, immunity, MAPK signaling and calcium homeostasis. Among these pathways, p38 MAPK and immunity have been previously related to longevity in numerous studies^27^,^28^,^29^,^30^,^31^. Our network analysis indicates these 4 identified pathways are highly interconnected. Collectively, our findings strongly support the notion that longevity is a polygenic trait influenced by a complex interplay of multiple genes and pathways. Interestingly, the three significant pathways (MAPK, immunity and calcium signaling) identified in our Han Chinese GWAS study were also most recently reconfirmed as significant pathways in the Danish longitudinal Epigenome-wide association study^36^. Our novel finding that the starch, sucrose and xenobiotic metabolism pathway is significantly associated with human longevity in Han Chinese is consistent with the previous animal model study in Drosophilia^34^. Together, these data strengthen our findings that combinations of SNPs or genes represented among GWAS associations could converge upon gene pathways and biological processes related to healthy aging.

Like other GWAS studies on longevity, the major limitation of the present study is a lack of physiologically relevant model systems for further biological and functional validation of our new findings. This study represents the largest GWAS of centenarians to date and provides a particularly useful reference for analysis of disease mitigation or prevention genotypes, pathways and biological mechanisms. We believe that sharing the findings of this study will inspire biologists who are interested in aging research to conduct functional studies of the various identified genetic variants and pathways associated with longevity.

The findings in the present study support the hypothesis that defensive mechanisms (such as immunity) and metabolism driven by diet in response to environmental stress may play key roles in longevity in the Han Chinese. Diet mediated mechanisms also suggest that genetic and epigenetic influences upon extreme survival can differ according to culture and ethnicity. These findings may provide novel insights for expanding longevity theories that have emphasized genes associated with maintenance of genome integrity (especially DNA repair) and fertility-related mechanisms^43^,^44^,^45^. Our new findings and previous studies about the reactive and adaptive nature of the immune system and about metabolism influenced by factors such as diet lead us to hypothesize that Han Chinese centenarians, who survived through the brutal past, may carry favorable defensive genotypes associated with longevity. Further studies are warranted for functional characterization of the new loci and interactive pathways to elucidate the underlying mechanisms for the observed associations with longevity.

## Methods

### M1. Samples and data source

DNA samples and data for the present GWAS are from the Chinese Longitudinal Healthy Longevity Surveys (CLHLS), which were conducted in 1998, 2000, 2002, 2005, 2008, 2011 and 2014 in a randomly selected half of the counties and cities in 22 out of 31 provinces in China. The CLHLS covers approximately 85% of the total population of China. We tried to interview all consented centenarians in the sampled counties and cities. For each centenarian interviewee, we recruited one nearby un-related middle-age control participant aged 40–59. “Nearby” is loosely defined – it could mean the same village or the same street if available, or in the same town or in the same sampled county or city^46^. In the present study, all of the DNA samples of the middle-age controls were collected in the same county/city or different county/city but in the same province, as that of the centenarians.

Phenotype data were collected in the CLHLS using internationally standardized questionnaires adapted to the Chinese cultural and social context^46^. Extensive evaluations of the data quality of the CLHLS, including assessments of mortality rate, proxy use, non-response rate, sample attrition, reliability and validity of major health measures, and the rates of logically inconsistent answers, have shown that the data from the CLHLS surveys are of good quality^47^. The genetic samples and data from CLHLS were successfully used in prior published studies on candidate genes and gene-environment interactions relevant to longevity^48^,^49^,^50^,^51^,^52^.

A wide variety of international and Chinese studies^53^,^54^ have confirmed that age reporting of the Han Chinese oldest-old aged 80 + , including centenarians, is reasonably accurate; this is due to the Han Chinese cultural tradition of memorizing one’s date of birth to determine dates of important life events such as engagement, marriage, starting to build a residential house, etc. The accuracy of age reporting in the CLHLS data was reconfirmed by an investigation, which compared various standard demographic indices of age reporting among the oldest-old and age distributions of centenarians between the CLHLS and comparable data from Sweden, Japan, England and Wales, Australia, Canada, the U.S., and Chile; this study concluded that the age reporting of the Han Chinese oldest-old, including centenarians, is as good as the average in developed countries^55^.

Note that the Han Chinese comprise about 93% of the total population in China, with 53 Chinese minority groups comprising 7% of the total population. The sample sizes of any minority group in the CLHLS data are too small for meaningful analysis, so we include Han Chinese samples only in the present study.

Descriptions of the samples and data sources of GWAS on longevity from the European Union Consortium and New England centenarians study (NECS) are given in the refs 4 and 10.

### M2. Genotyping and quality controls

After DNA extraction, all of the 2,578 centenarians and 2,387 middle-age controls were genotyped using the Illumina HumanOmniZhongHua-8 BeadChips, which was created by strategically selecting optimized tag SNP content from all three HapMap phases and the 1000 Genomes Project (1 kGP). It allows profiling of 900,015 SNPs per sample, including 600 k SNPs of common variants (MAF ≥ 5%), 290 k SNPs of rare variants (MAF < 5%) and 10 k SNPs existing only among Chinese and other Asian populations. In other words, 98.9% of the 900k SNPs of the Illumina HumanOmniZhongHua-8 BeadChip are internationally compatible, with 1.1% specific to Chinese and Asian populations; this provides coverage of about 81% of common variants at r^2^ > 0.8 with MAF ≥ 5%, and about 60% of rare variants at r^2^ > 0.8 with MAF < 5%. Our selection of this chip represents a state-of-the-art choice for GWAS in Asian populations to maximize international compatibility.

We randomly arranged cases and controls during sample batches, with negative controls (buffer water) and positive controls (YanHuang samples). For the sample filtering, 339 individuals for whom the genotypes were generated with a call rate less than 95% were excluded. We also computed identity-by-state probabilities for all subjects to search for any possible duplicates and kinship-related individuals among the samples, using PLINK (1.06)^16^. The 134 individuals who had identity-by-state probabilities with PI_HAT > 0.25 were excluded based on the IBD analysis implemented in PLINK.

To complete the sample filtering data quality control procedures and investigate population stratification in our GWAS samples, we performed principal components analysis (PCA) to evaluate genomic correlations in our dataset on the basis of pairwise identity by state (IBS) for all of the successfully genotyped samples using PLINK (1.06)^16^. We used the filter option (MAF > 0.01 and genotyping rate > 0.9) as discussed above; about 80% of the SNPs were used to calculate IBS. Our principle components analysis (PCA) showed that 9 individuals (all from Fujian province) were genetically separated from the total population of the 22 provinces (including Fujian) where the CLHLS was conducted, so these 9 individuals were excluded from our GWAS. Based on the PCA results, we also excluded 6 individuals who had serious deviations from the genetically-derived geographic membership in Southern or Northern populations.

After excluding these 15 outliers, our PCA showed that the cases and controls in this study were of Han Chinese ancestry and were well matched, without evidence of gross population stratification. In general, cases and controls were not separated on the basis of the first two principal components, and the cases and controls are evenly distributed in clusters of PC1 vs. PC2, PC1 vs. PC3 and PC2 vs. PC3 (see Supplementary Fig. 1). We computed the inflation factor (λ), a metric describing genome-wide inflation in association statistics^56^, both before or after correcting for stratification using PLINK (1.06)^16^. Values of λ after correcting along 0, 1, 2, 3, or 4 eigenvectors are 1.109, 1.0241, 1.0235, 1.0226, or 1.0221, respectively, demonstrating that the correction is important and the top two eigenvectors correct nearly all of the stratification that can be corrected using the 4 eigenvectors. Consequently, in the subsequent GWAS regression models, we adjusted for the top two eigenvectors to minimize the effects of population stratification, following the approach adopted in the refs 54 and 57.

Finally, after various stages of sample filtering, 2,178 centenarians (mean age 102.7 ± 3.49 (SD)) and 2,299 middle-age controls (mean age 48.4 ± 7.44 (SD)) were included in the subsequent GWAS dataset (Supplementary Table 1). We also conducted quality-control filtering of the GWAS data from the total 4,477 individuals. Following the standard adopted in the published studies of GWAS using the same 900 k SNPs Illumina HumanOmniZhongHua-8 BeadChips^58^ as the one used in present study, SNPs with call rates of less than 90% were removed from our GWAS dataset; SNPs were also excluded if they had a MAF less than 1% or if there was significant deviation from Hardy-Weinberg equilibrium in the samples, defined as *P* < 10^−5^. SNPs on the X and Y chromosomes and mitochondria were removed from the initial statistical analysis. Only 242 out of 743 (~30%) GWAS conducted from 2005 to 2011 considered chromosome X in their analyses according to the recent report of Wise *et al*.^59^. Wise *et al*. discussed several reasons for exclusion of chromosome X, including a lower proportion of genes on chromosome X and a lower coverage of chromosome X on current genotyping platforms compared with autosomal coverage. They also described a number of technical hurdles that might add to the reluctance to include chromosome X in a GWAS, including complications in genotype calling, imputation, selection of test statistics, and the lack of readily available implementations. Accordingly, we excluded the chromosome X. After quality filtering and cleaning, 818, 084 genotyped SNPs remained for association analysis in our final GWAS dataset.

### M3. Imputation

To further increase genome coverage, we performed imputation analysis to infer the genotypes of all SNPs (MAF ≥ 0.01) using IMPUTE software version 2^60^ and the 1000 Genomes Project integrated phase 1 release as reference panel. SNPs with a quality score (Rsq) of <0.9 were discarded before analysis. After standard GWAS quality-control filtering for subjects and SNPs as described above, we obtained data for 5, 595, 657 SNPs (818, 084 genotyped SNPs and 4, 777, 573 imputed SNPs) in 2, 178 centenarians cases and 2, 299 middle-age controls for the subsequent GWAS analyses.

### M4. Stratifying Southern and Northern regions for our discovery-evaluation analysis

China is considered to have geographically and traditionally distinct Southern and Northern regions that have existed for a few thousand years, although there is no legal or official administrative meaning to this division. As reviewed in Xu *et al*.^15^, previous analyses of anthropological, anatomical, linguistic, and genetic data (including classic markers, microsatellite DNA markers, mtDNA, and Y chromosome SNP markers) have consistently suggested the presence of a significant boundary between the Southern and Northern populations in China. Among the 22 provinces where the CLHLS was conducted, Liaoning, Jilin, Heilongjiang, Hebei, Beijing, Tianjin, Shanxi, Shandong, Shaanxi, Jiangsu, Anhui and Henan belong to the geographically and traditionally defined Northern region; Shanghai, Zhejiang, Fujian, Jiangxi, Hubei, Hunan, Guangdong, Sichuan, Guangxi, Chongqing, and Hainan belong to the geographically and traditionally defined Southern region.

Our principle components analysis (described in section M2 above) also genetically confirmed the geographic and traditional cultural division between Southern and Northern regions in the Han Chinese GWAS dataset (Supplementary Fig. 2). Thus, we divided the entire sample into two independent GWAS datasets of Southern and Northern regions for our discovery-evaluation analysis. The Southern region dataset consists of 1,063 centenarians and 887 middle-age controls and the Northern region dataset consists of 1,115 centenarians and 1,412 middle-age controls (Supplementary Table 1). Given the nature of our population analysis and due to the fact that all of the samples analyzed in this study belong to the same ethnic group of Han Chinese living in one country with the same culture, we also performed a combined analysis comparing all centenarians with all controls. The genomic inflation factors (λ) in the Southern and Northern datasets and the combined dataset were 1.022, 1.010 and 1.022, respectively, suggesting that the association test statistics conformed to the underlying null distribution and would not require further adjustment for genomic control.

### M5. A bi-directional discovery-evaluation strategy

In the classic uni-directional discovery-replication approach, the entire sample is divided into two datasets with one dataset used as discovery and the other dataset as the replication or valuation. The top SNPs found in the discovery stage using a pre-determined *P* value threshold are analyzed in the evaluation dataset. The SNPs with a *P* value lower than the threshold (e.g. *P* < 1.0 × 10^−4^) in the discovery stage and nominal significance (e.g. *P* < 0.05) in the evaluation stage are identified as the significant/replicated SNPs, which are the final results of the analysis. This classic uni-directional discovery-evaluation approach is especially useful when the expensive GWAS serves as the first stage of discovery and the much less expensive second stage of evaluation genotypes only the top SNPs with a *P* value lower than the threshold found in the first stage. However, when analyzing two available independent GWAS datasets, the uni-directional discovery-evaluation approach of assigning one GWAS dataset as discovery and another GWAS dataset as evaluation would have a higher false-negative rate, missing a substantial number of significant/replicated SNPs which have a p-value higher than the threshold and lower than the nominal significance level in the discovery GWAS dataset but reach the threshold significance level in the evaluation GWAS dataset.

To avoid the high false-negative rate and to fully utilize all information in the two independent Southern and Northern GWAS datasets available to us, we applied a novel bi-directional discovery-evaluation strategy^17^ to search for consistent associations of SNPs with longevity. More specifically, the two GWAS datasets from Southern and Northern regions were analyzed with a parallel and bi-directional procedure: one is assigned as a discovery dataset and another as an evaluation dataset, and vice versa^17^. In our case, we first assigned and analyzed the Southern region GWAS dataset as discovery and Northern region GWAS dataset as evaluation, and then analyzed the Northern region GWAS dataset as discovery and Southern region GWAS dataset as evaluation (see Fig. 1). In this way, we capture all SNPs associated with longevity with a p-value lower than the threshold in the discovery stage and nominal significance in the evaluation stage.

The recent literature on GWAS discovery-evaluation analysis indicate that researchers defined a *priori* discovery threshold of *P* < 10^−5^ or *P* < 10^−4^, or *P* < 10^−3^, depending on the circumstances of the research topics and the characteristics of the datasets used^61^,^62^,^63^. In the present study of GWAS on longevity, we use exceptionally long-lived centenarians as cases and middle-aged adults as controls. Furthermore, we seek to identify SNPs which may individually have small effects, but may have large and important joint effects on the extremely complex trait of exceptional longevity. Thus, we believe that selecting the modest *a priori* discovery threshold of *P* < 10^−4^ is a reasonable choice. More specifically, we defined the *a priori* discovery threshold of *P* < 10^−4^ in the discovery stage in our bi-directional discovery-evaluation analyses using the independent GWAS datasets of Southern and Northern rejoins of China, and in further international comparative analysis among the longevity GWAS datasets of the CLHLS, European Union and New England. We used a nominal significance level of p < 0.05 in the evaluation stage, following the usually adopted practice.

### M6. Case-control study design and the statistical analysis

The logistic regression approach (named as “case-control association analysis on longevity”) or the “Fixed-Attributes Dynamics (FAD) Method”^64^ and its extension^48^ is often used to estimate the associations between genetic variants and longevity, through comparing long-lived individuals and middle-age controls^4^,^10^,^11^,^51^,^52^. In fact, the “case-control association analysis on longevity” approach uses the same basic idea of FAD and produces exactly the same results of estimates as FAD does^48^.

The method of case-control association analysis (or FAD) on longevity, which uses long-lived individuals as cases and ethnically/geographically matched middle-age persons as controls, is based on fundamental demographic insight that the prevalence of a genetic variant in a population can change with age even though no individuals can change their fixed attribute of the genetic variant, and that therefore much can be learned about the impact of the genetic variant on longevity. Ideally, we would use complete cohort data to compare the distributions of the genetic variants of members of the same cohort at two points of time in their life span: when they were young versus when some of them reach age 100 + , while all of the other cohort members died before age 100. However, such long-term and complete follow-up GWAS or candidate gene genotype data for the members of the same birth cohort at young ages and ages 100 + are not currently available for any population. Thus, various previously published association studies on longevity used cross-sectional datasets to compare long-lived individuals (centenarians and/or nonagenarians) as cases and middle-aged as controls, observed in the same period. Such cross-sectional cases/controls analyses are based on two assumptions: (1) the initial distribution of the genetic variants does not differ substantially between the long-lived and middle-age cohorts, which is generally reasonable, because the basic genetic structure would not change substantially in about 50 years within the same ethnic population. (2) The basic genetic profiles of the non-migrants do not differ substantially from those migrants who are of the same ethnicity as non-migrants. This assumption is also generally reasonable, especially for studies of Han Chinese because China received remarkably few immigrants of other ethnicities in the past decades. With these two assumptions, one may intuitively understand that the proportion of genetic variants which are positively (or negatively) associated with longevity are significantly higher (or lower) among the centenarians, compared to the middle-aged controls, because those who have the favorite genetic profile have better chance to survive to age 100 + , while those with less favorite genetic profile could not reach age 100. Such an intuitive mechanism based on the two assumptions outline above has been proven mathematically^48^.

Note that in the middle aged controls, selective mortality is still very low and on the other hand it is impossible for natural selection to change the gene pool within a short period of several decades. Thus, genotype frequency in the middle-aged controls could well represent population genotypes. However, the findings based on the study design of using centenarians as cases and middle-aged as controls may be driven by the gene-environment interactions that lead to selective mortality for the centenarians. For example, the Chinese centenarians survived the brutal mortality regimes of the past when famine, wars, and starvation operated on their large birth cohorts. This may imply that these long-lived individuals who successfully passed through the hard life conditions and high mortality of earlier decades may carry genotypes that interact with environmental factors such as life stress, certain diet or behavior, which lead to exceptional longevity. Thus, the genotypes associated with longevity identified through comparing centenarians and middle-age controls may serve as candidates for further studies on effects of gene-environment interactions on longevity and health to determine whether they are found in other populations and cohorts that have been subjected to similar or different environmental factors.

We performed GWAS analysis using logistic regression as implemented in PLINK (1.06)^16^. To minimize the effects of population stratification, we adjusted for the top two eigenvectors, which corrected nearly all of the stratification that can be corrected^57^. In the combined data analysis, we also adjusted for the sex and geographic stratification of Southern and Northern regions. In addition, we performed the meta-analysis, treating the two independent datasets from Southern and Northern regions of China as two groups and employing the METAL software^65^.

For the SNPs associated with longevity and replicated in the datasets of the Southern and Northern regions, we also conducted gender-specific analyses and tested whether the differences in the effect sizes measured by the odds ratio were statistically significant between the two genders. We used Woolf’s test^66^ to calculate *P* values to measure the gender differences in association with longevity (Supplementary Table 2). Woolf’s test is a method for testing the heterogeneity of 2 × 2 contingency tables over multiple strata, which is sensitive when the odds ratios (OR) of two genders are in opposite directions (the allele is protective for one sex and risky for the other), or in the same direction but of significantly different magnitude of effect size.

The methods of statistical analyses using the GWAS datasets from EU longevity genetics consortium and NECS were described in the refs 4 and 10, respectively^4^,^10^.

### M7. Pathway analysis

High-throughput single-SNP association analysis per se does not directly produce biologically functional findings because SNPs and genes work in an intricate network of interactions through biological pathways. In addition, each of the significant SNPs normally has a small effect on a complex trait such as longevity. Thus, pathway analysis is an important follow-up for GWAS studies to provide mechanistic insight about the underlying biology of the phenotype of interest. By identifying well-annotated pathways that map to the lists of significant genes identified in a GWAS, biochemical hypotheses can be enumerated and tested^67^. Thus, to further investigate the large number of SNPs produced by our GWAS in the context of biological processes, we conducted pathway and network analysis. We applied an algorithm known as improved gene set enrichment analysis for GWAS (i-GSEA4GWAS) to place variants associated with longevity within curated pathways and functional categories^68^. SNPs with p < 0.05 in our combined GWAS dataset were mapped to genes if within a 20 kb distance (upstream or downstream). For a given SNP, if multiple genes were located within this range, the closest gene was selected and assigned the association *P* value. Since multiple SNPs can map to the same gene, a SNP label permutation was used to reduce biases caused by larger loci having a disproportionately higher number of SNPs. We did not mask the non-MHC/xMHC region and set the gene size to minimum 10 and maximum 200, based on the filter criteria recommended by Ramanan *et al*.^69^ The program uses the list of SNP-mapped genes to filter the collection of pathways/gene sets to obtain candidate pathways/gene sets. In total, 127, 092 SNPs associated with longevity with a p < 0.05 were used and they were mapped to 8,085 genes. These were limited to curated pathways derived from multiple resources such as KEGG^70^, REACTOME^71^ and BioCarta (http://www.biocarta.com/), and functional annotations extracted from the Gene Ontology database^72^. A modified version of the GSEA procedure was performed, adjusted for multiple testing using false discovery rate (FDR), and pathways/gene sets with corrected p-value < 0.05 and FDR < 0.05 regarded as associated with longevity traits^68^.

### M8. Gene interaction network analysis

To investigate whether and how the pathways may interact to affect longevity, we performed gene interaction network analysis. We first sorted the genes which were highly-represented across the 25 enriched pathways. Then we selected 35 genes which were constituents of 2 or more enriched pathways, and used the human protein-chemical interactions database STITCH 4.0^73^ to investigate the interaction network among significant genes and among genes and chemicals. The STITCH database accumulates data from multiple sources and contains a confidence value for each interaction. The confidence scores range between 0 and 1, with a default cutoff value of 0.15. To ensure that the results of our gene interaction network analysis attain a high confidence level, we generated evaluation datasets for STITCH in which we required a confidence score of at least 0.4 (median value)^73^.

### M9. Expression Quantitative Trait Loci (eQTL) analysis

We examined the potential effects of the SNPs, identified as associated with longevity at a suggestive significance level in our GWAS, on gene expression, using the three publicly available expression Quantitative Trait Loci (eQTL) databases: (1) the Genotype-Tissue Expression (GTEx) data derived from multiple cell and tissue types (lymphoblastoid cell lines, brain tissue and human fibroblasts)^74^; (2) SeeQTL database from the University of North Carolina at Chapel Hill^75^; and (3) eQTL resources from the Gilad/Pritchard group at University of Chicago (http://eqtl.uchicago.edu/Home.html). In total, 127, 092 SNPs with *P* < 0.05 in the combined datasets of our GWAS were included to examine their effects on gene expression in using these three eQTL database resources.

### M10. Statements of Approval for Human Subjects Informed Consent and in accordance with relevant guidelines

The Research Ethics Committees of Duke University and Peking University granted approval for the Protection of Human Subjects for the Chinese Longitudinal Healthy Longevity Survey, including collection of DNA sample used for present study. The survey respondents who contributed their DNA samples gave informed consent before participation. All of the GWAS experiments and methods of analyses in present study were performed in accordance with relevant guidelines and regulations.

## Additional Information

**How to cite this article**: Zeng, Y. *et al*. Novel loci and pathways significantly associated with longevity. *Sci. Rep*. **6**, 21243; doi: 10.1038/srep21243 (2016).

## Supplementary Material

Supplementary Information

## Figures and Tables

**Table 1:** The 11 independent loci associated with longevity at p < 10^−4^ in the discovery and at least a nominal significance (p < 0.05) in the evaluation, using the independent GWAS datasets of Southern and Northern regions of China as discovery/evaluation.

SNP	Chr.	Position	Nearest gene	Coded/noncoded allele	*Southern region of China*			*Northern region of China*			Southern-Northern Combined			Metaanalysis
					MAF (case/control)	P	Oddsratio	MAF (case/control)	P	Oddsratio	MAF (case/control)	P	Oddsratio	P
rs2069837	7	22768027	*IL6*(intronic)	G/A	0.018/0.033	5.98E-03	0.582	0.086/0.134	1.00E-06	0.64	0.053/0.095	1.80E-09	0.61	4.05E-08
rs2440012	13	19440123	*ANKRD20A9P*(nc_exonic)	G/C	0.050/0.092	1.38E-06	0.506	0.057/0.079	2.26E-03	0.69	0.054/0.084	3.73E-08	0.602	4.89E-08
rs145672791	21	14750023	*MIR3156-3*(28 kb downstream)	A/G	0.003/0.011	5.08E-03	0.267	0.004/0.022	9.88E-06	0.203	0.004/0.018	8.95E-08	0.219	2.34E-07
rs61856137	10	5087978	*AKR1C2*(27 kb upstream)	T/G	0.019/0.032	9.85E-03	0.572	0.040/0.070	1.56E-05	0.549	0.029/0.056	1.60E-07	0.544	7.54E-07
rs2704588	4	89849772	*FAM13A*(intronic)	C/T	0.004/0.013	4.32E-03	0.289	0.005/0.021	3.26E-05	0.237	0.004/0.018	2.38E-07	0.248	5.63E-07
rs1487614	4	42269480	*BEND4*(114 kb upstream)	T/C	0.112/0.146	1.85E-03	0.738	0.103/0.141	8.13E-05	0.707	0.107/0.143	2.87E-07	0.716	5.30E-07
rs10934524	3	96150160	*EPHA6*(383 kb upstream)	T/C	0.453/0.384	2.97E-05	1.354	0.470/0.431	4.76E-03	1.192	0.462/0.413	5.33E-07	1.266	1.16E-06
rs57681851	4	2290698	*ZFYVE28*(intronic)	G/T	0.187/0.136	7.05E-05	1.448	0.155/0.128	7.75E-03	1.256	0.170/0.131	1.83E-06	1.348	3.78E-06
rs7213812	17	31448649	*ASIC2*(intronic)	C/A	0.216/0.161	1.36E-05	1.45	0.176/0.152	2.84E-02	1.182	0.196/0.155	6.33E-06	1.29	6.25E-06
rs9568833	13	53827016	*OLFM4*(200 kb downstream)	T/C	0.145/0.193	7.85E-05	0.712	0.144/0.168	2.46E-02	0.836	0.144/0.177	1.77E-05	0.778	1.75E-05
rs405509	19	45408836	*APOE*(200 bp upstream)	G/T	0.374/0.316	7.92E-05	1.32	0.308/0.279	2.56E-02	1.148	0.341/0.293	3.64E-05	1.21	1.85E-05

Note: The SNPs rs57681851 and rs7213812 were imputed, while the other 9 SNPs listed in this Table were genotyped. MAF; minor allele frequency.

## References

[b1] Hjelmborg J. . Genetic influence on human lifespan and longevity. Hum Genet. 119, 312–321 (2006). 16463022 10.1007/s00439-006-0144-y

[b2] Tan Q. . Analyzing age-specific genetic effects on human extreme age survival in cohort-based longitudinal studies. European Journal of Human Genetics. 21, 451–454 (2013). 22892531 10.1038/ejhg.2012.182 PMC3598313

[b3] Sebastiani P. . Increasing Sibling Relative Risk of Survival to Older and Older Ages and the Importance of Precise Definitions of “Aging,” “Life Span,” and “Longevity”. J. Gerontol. A. Biol. Sci. Med. Sci. doi: 10.1093/gerona/glv020 (2015). PMC4757962 25814633

[b4] Sebastiani P. . Genetic signatures of exceptional longevity in humans. PLoS One 7, e29848 (2012). 22279548 10.1371/journal.pone.0029848 PMC3261167

[b5] Christensen K. . Exceptional longevity does not result in excessive levels of disability. Proc. Natl. Acad. Sci. USA 105, 13274–13279 (2008). 18711139 10.1073/pnas.0804931105 PMC2517602

[b6] Hitt R., Young-Xu Y. & Perls T. Centenarians: The older you get, the healthier you’ve been. Lancet, 354, 652 (1999). 10466675 10.1016/S0140-6736(99)01987-X

[b7] Andersen S., Sebastiani P., Deworkis D., Feldman L. & Perls T. Health span approximates life span amongst many supercentenarians: Compression of morbidity at the approximate limit of life span. J Gerontol Biol Sci. 67, 395–405 (2012). 10.1093/gerona/glr223 PMC3309876 22219514

[b8] Zeng Y. . Health consequences of familial longevity influence among the Chinese elderly. J. Gerontol. A. Biol. Sci. Med. Sci. 68, 473–82 (2013). 23064818 10.1093/gerona/gls203 PMC3593617

[b9] Terry D. F. . Reduced all-cause, cardiovascular and cancer mortality in centenarian offspring. J. Amer. Geriatr. Soc. 52, 2074–2076 (2004). 15571545 10.1111/j.1532-5415.2004.52561.x

[b10] Deelen J. . Genome-wide association meta-analysis of human longevity identifies a novel locus conferring survival beyond 90 years of age. Hum. Mol. Genet. 23, 4420–4432 (2014). 24688116 10.1093/hmg/ddu139 PMC4103672

[b11] Newman A. B. . A meta-analysis of four genome-wide association studies of survival to age 90 years or older: the Cohorts for Heart and Aging Research in Genomic Epidemiology Consortium. J. Gerontol. A Biol. Sci. Med. Sci. 65, 478–487 (2010). 20304771 10.1093/gerona/glq028 PMC2854887

[b12] Jeune B. In search of the first centenarians. In: Jeune B. & Vaupel J. (eds.) Exceptional longevity: from prehistory to the present. Odense University Press. 11–24 (1995).

[b13] Xu S. & Jin L. A Genome-wide Analysis of Admixture in Uyghurs and a High-Density Admixture Map for Disease-Gene Discovery. Am. J. Hum. Genet. 83, 322–336 (2008). 18760393 10.1016/j.ajhg.2008.08.001 PMC2556439

[b14] Weir B. S. & Hill W. G. Estimating F-statistics. Annu. Rev. Genet. 36, 721–750 (2002). 12359738 10.1146/annurev.genet.36.050802.093940

[b15] Xu S. . Genomic dissection of population substructure of Han Chinese and its implication in association studies. Am. J. Hum. Genet. 85, 762–774 (2009). 19944404 10.1016/j.ajhg.2009.10.015 PMC2790582

[b16] Purcell S. . PLINK: a tool set for whole-genome association and population-based linkage analyses. Am. J. Hum. Genet. 81, 559–575 (2007). 17701901 10.1086/519795 PMC1950838

[b17] Jia P. . Network-assisted investigation of combined causal signals from genome-wide association studies in schizophrenia. PLoS Comput Biol. 8, e1002587 (2012). 22792057 10.1371/journal.pcbi.1002587 PMC3390381

[b18] Soerensen M. . Evidence from case–control and longitudinal studies supports associations of genetic variation in APOE, CETP, and IL6 with human longevity. Age 35, 487–500 (2013). 22234866 10.1007/s11357-011-9373-7 PMC3592963

[b19] Christiansen L. . Paraoxonase 1 polymorphisms and survival. Eur J Hum Genet. 12, 843–847 (2004). 15241482 10.1038/sj.ejhg.5201235

[b20] Bonafè M. . A gender—dependent genetic predisposition to produce high levels of IL-6 is detrimental for longevity. Eur. J. Immunol. 31, 2357–2361 (2001). 11500818 10.1002/1521-4141(200108)31:8<2357::aid-immu2357>3.0.co;2-x

[b21] Cohen H. J., Harris T. & Pieper C. F. Coagulation and activation of inflammatory pathways in the development of functional decline and mortality in the elderly. Am. J. Med. 114, 180–187 (2003). 12637131 10.1016/s0002-9343(02)01484-5

[b22] Yang J. . Common SNPs explain a large proportion of the heritability for human height. Nat. Genet. 42, 565–569 (2010). 20562875 10.1038/ng.608 PMC3232052

[b23] Broer L. . GWAS of Longevity in CHARGE Consortium Confirms APOE and FOXO3 Candidacy. J. Gerontol. A. Biol. Sci. Med. Sci. 70, 110–118 (2015). 25199915 10.1093/gerona/glu166 PMC4296168

[b24] Zhang K. . i-GSEA4GWAS: a web server for identification of pathways/gene sets associated with traits by applying an improved gene set enrichment analysis to genome-wide association study. Nucleic Acids Res. 38, W90–W95 (2010). 20435672 10.1093/nar/gkq324 PMC2896119

[b25] Lambert J. C. . Meta-analysis of 74, 046 individuals identifies 11 new susceptibility loci for Alzheimer’s disease. Nat. Genet. 45, 1452–1458 (2013). 24162737 10.1038/ng.2802 PMC3896259

[b26] Karch C. M. . Alzheimer’s disease genetics: from the bench to the clinic. Neuron. 83, 11–26 (2014). 24991952 10.1016/j.neuron.2014.05.041 PMC4120741

[b27] Asai T. . MAP kinase signaling cascade in Arabidopsis innate immunity. Nature 415, 977–983 (2002). 11875555 10.1038/415977a

[b28] Naumova E., . Immunogenetics of ageing. Int. J. Immunogenet. 38, 373–381 (2011). 21726414 10.1111/j.1744-313X.2011.01022.x

[b29] Apfeld J. . The AMP-activated protein kinase AAK-2 links energy levels and insulin-like signals to lifespan in C. elegans. Genes. Dev. 18, 3004–3009 (2004). 15574588 10.1101/gad.1255404 PMC535911

[b30] Anisimov V. N. . Metformin slows down aging and extends life span of female SHR mice. Cell Cycle 7, 2769–2773 (2008). 18728386 10.4161/cc.7.17.6625

[b31] Yao Y. . Proteasomes, Sir2, and Hxk2 Form an Interconnected Aging Network That Impinges on the AMPK/Snf1-Regulated Transcriptional Repressor Mig1. PLoS Genet. 11, e1004968 (2015). 25629410 10.1371/journal.pgen.1004968 PMC4309596

[b32] Li X. . Integrated genomic approaches identify major pathways and upstream regulators in late onset Alzheimer’s disease. Sci. Rep. 5, 12393 (2015). 26202100 10.1038/srep12393 PMC4511863

[b33] Semchyshyn H. M. . Fructose and glucose differentially affect aging and carbonyl/ oxidative stress parameters in Saccharomyces cerevisiae cells. Carbohydr. Res. 346, 933–938 (2011). 21459368 10.1016/j.carres.2011.03.005

[b34] Troen A. M. . Lifespan modification by glucose and methionine in Drosophila melanogaster fed a chemically defined diet. Age 29, 29–39 (2007). 19424828 10.1007/s11357-006-9018-4 PMC2267680

[b35] Zhang H. . Calcium signaling, excitability, and synaptic plasticity defects in mouse model of Alzheimer’s disease. J. Alzheimers. Dis. 45, 561–580 (2015). 25589721 10.3233/JAD-142427 PMC4814213

[b36] Tan Q. H., Christensen k. & Christiansen L. Epigenetic drift in the aging genome: a ten-year follow-up in an elderly cohort of twins. Paper presented at the International Conference on “Interdisciplinary Research on Long-term Care and Healthy Aging”, May 22-23, 2015, Hangzhou, China. (2015).

[b37] He K. . Dynamic regulation of genetic pathways and targets during aging in Caenorhabditis elegans. AGING 6, 215–230 (2014). 24739375 10.18632/aging.100648 PMC4012938

[b38] Kuhn M. . STITCH 4: integration of protein-chemical interactions with user data. Nucleic. Acids. Res. 42, D401–D407 (2014). 24293645 10.1093/nar/gkt1207 PMC3964996

[b39] Troemel E. R. . p38 MAPK regulates expression of immune response genes and contributes to longevity in C. elegans. PLoS Genet. 2, e183 (2006). 17096597 10.1371/journal.pgen.0020183 PMC1635533

[b40] Boyle A. P. . Annotation of Functional Variation in Personal Genomes Using RegulomeDB. Genome Research 22, 1790–1797 (2012). 22955989 10.1101/gr.137323.112 PMC3431494

[b41] Montgomery S. B. . Transcriptome genetics using second generation sequencing in a Caucasian population. Nature 464, 773–777 (2010). 20220756 10.1038/nature08903 PMC3836232

[b42] Rebeck G. W. . Reduced apolipoprotein epsilon 4 allele frequency in the oldest old. Alzheimer’s patients and cognitively normal individuals. Neurology 44, 1513–1516 (1994). 8058160 10.1212/wnl.44.8.1513

[b43] Kirkwood T. B. L. & Kowald A. The free‐radical theory of ageing–older, wiser and still alive. Bioessays 34, 692–700 (2012). 22641614 10.1002/bies.201200014

[b44] Baudisch A. & Vaupel J. W. Getting to the root of aging. Science 338, 618 (2012). 23118175 10.1126/science.1226467 PMC3705936

[b45] Sun F. . Extended maternal age at birth of last child and women’s longevity in the Long Life Family Study. Menopause 22, 26–31 (2015). 24977462 10.1097/GME.0000000000000276 PMC4270889

[b46] Zeng Y. Towards Deeper Research and Better Policy for Healthy Aging—Using the Unique Data of Chinese Longitudinal Healthy Longevity Survey. China Economic Journal 5, 131–149 (2012). 24443653 10.1080/17538963.2013.764677 PMC3893304

[b47] Goodkind D. Review on the book Healthy Longevity in China: Demographic, Socioeconomic, and Psychological Dimensions. Population Studies 63, 1–7 (2009).

[b48] Zeng Y. . Effects of FOXO genotypes on longevity: a biodemographic analysis. J. Gerontol. A Biol. Sci. Med. Sci. 65, 1285–1299 (2010). 20884733 10.1093/gerona/glq156 PMC2990269

[b49] Zeng Y. . Interactions between Social/behavioral factors and ADRB2 genotypes may be associated with health at advanced ages in China. BMC geriatrics 13, 91–91 (2013). 24016068 10.1186/1471-2318-13-91 PMC3846634

[b50] Zeng Y. . GxE Interactions between FOXO Genotypes and Tea Drinking Are Significantly Associated with Cognitive Disability at Advanced Ages in China. J. Gerontol. A Biol. Sci. Med. Sci. 70(4), 426–433. doi: 10.1093/gerona/glu060. (2015). 24895270 PMC4447795

[b51] Li Y. . Genetic association of FOXO1A and FOXO3A with longevity trait in Han Chinese populations. Human molecular genetics 18, 4897–4904 (2009). 19793722 10.1093/hmg/ddp459 PMC2790334

[b52] Zhao L. . Common genetic variants of the β2-adrenergic receptor affect its translational efficiency and are associated with human longevity. Aging Cell, 11, 1094–1101 (2012). 23020224 10.1111/acel.12011 PMC3633790

[b53] Coale A. J. & Li S. The effect of age misreporting in China on the calculation of mortality rates at very high ages. Demography 28, 293–301 (1991). 2070900

[b54] Wang Z. . Age validation of Han Chinese centenarians. GENUS 54, 123–141 (1998). 12321975

[b55] Zeng Y. & Gu D. Reliability of age reporting among the Chinese oldest-old in the CLHLS datasets. In: Zeng Y. . (eds.). Healthy longevity in China: Demographic, socioeconomic, and psychological dimensions. pp. 61–80. Dordrecht, The Netherlands: Springer Publisher, (2008).

[b56] Devlin B. & Roeder K. Genomic control for association studies. Biometrics 55, 997–1004 (1999). 11315092 10.1111/j.0006-341x.1999.00997.x

[b57] Price A. L. . Discerning the ancestry of European Americans in genetic association studies. PLoS genetics 4, e236 (2008). 18208327 10.1371/journal.pgen.0030236 PMC2211542

[b58] Chen Y. . Common variants near ABCA1 and in PMM2 are associated with primary open-angle glaucoma. Nature genetics 46, 1115–1119 (2014). 25173107 10.1038/ng.3078

[b59] Wise A. L. . eXclusion: toward integrating the X chromosome in genome-wide association analyses. Am. J. Hum. Genet. 92, 643–647 (2013). 23643377 10.1016/j.ajhg.2013.03.017 PMC3644627

[b60] Marchini J. . A new multipoint method for genome-wide association studies by imputation of genotypes. Nat. Genet. 39, 906–913 (2007). 17572673 10.1038/ng2088

[b61] Chen Y. . Common variants near ABCA1 and in PMM2 are associated with primary open-angle glaucoma. Nature Genetics 46, 1115–1119 (2014). 25173107 10.1038/ng.3078

[b62] International Multiple Sclerosis Genetics Consortium (IMSGC). Analysis of immune-related loci identifies 48 new susceptibility variants for multiple sclerosis. Nat Genet. 45, doi: 10.1038/ng.2770 (2013). PMC3832895 24076602

[b63] Saxena R. . Genome-wide association study identifies a novel locus contributing to type 2 diabetes susceptibility in Sikhs of Punjabi origin from India. Diabetes. 62, 1746–55 (2013). 23300278 10.2337/db12-1077 PMC3636649

[b64] Zeng Y. & Vaupel J. W. Association of late childbearing with healthy longevity among the oldest-old in China. Population Studies. 58, 37–53 (2004). 15204261 10.1080/0032472032000175437

[b65] Willer C. J., Li Y. & Abecasis G. R. METAL: fast and efficient meta-analysis of genomewide association scans. Bioinformatics. 26, 2190–2191 (2010). 20616382 10.1093/bioinformatics/btq340 PMC2922887

[b66] Woolf B. On estimating the relation between blood group and disease. Ann. Hum. Genet. 19, 251–253 (1955). 14388528 10.1111/j.1469-1809.1955.tb01348.x

[b67] Khatri P. . Ten years of pathway analysis: current approaches and outstanding challenges. PLoS Comput Biol. 8, e1002375 (2012). 22383865 10.1371/journal.pcbi.1002375 PMC3285573

[b68] Zhang K. . i-GSEA4GWAS: a web server for identification of pathways/gene sets associated with traits by applying an improved gene set enrichment analysis to genome-wide association study. Nucleic Acids Res. 38, W90–W95 (2010). 20435672 10.1093/nar/gkq324 PMC2896119

[b69] Ramanan V. K. . Genome-wide pathway analysis of memory impairment in the Alzheimer’s Disease Neuroimaging Initiative (ADNI) cohort implicates gene candidates, canonical pathways, and networks. Brain Imaging Behav. 6, 634–648 (2012). 22865056 10.1007/s11682-012-9196-x PMC3713637

[b70] Kanehisa M. & Goto S. KEGG: Kyoto Encyclopedia of Genes and Genomes. Nucleic Acids Res. 28, 27–30 (2000). 10592173 10.1093/nar/28.1.27 PMC102409

[b71] Joshi-Tope G. . Reactome: a knowledgebase of biological pathways. Nucleic Acids Res. 1, D428–D432 (2005). 15608231 10.1093/nar/gki072 PMC540026

[b72] Ashburner M. . Gene ontology: tool for the unification of biology. Nat. Genet. 25, 25–29 (2000). 10802651 10.1038/75556 PMC3037419

[b73] Kuhn M. . STITCH 4: integration of protein-chemical interactions with user data. Nucleic. Acids. Res. 42, D401–D407 (2014). 24293645 10.1093/nar/gkt1207 PMC3964996

[b74] The Genotype-Tissue Expression Consortium. The Genotype-Tissue Expression (GTEx) project. Nat Genet. 45, 580–585 (2013). 23715323 10.1038/ng.2653 PMC4010069

[b75] Sullivan P. F. & Wright F. A. seeQTL: A searchable database for human eQTLs. Bioinformatics 28, 451–452 (2012). 22171328 10.1093/bioinformatics/btr678 PMC3268245

