# Supplementary material for: Novel loci and pathways significantly associated with longevity
Source: Sci Rep. 2016 Feb 25;6:21243. doi: 10.1038/srep21243 (PMC4766491; doi:10.1038/srep21243)
Supplement: Supplementary Information [file srep21243-s1.pdf]

Supplementary Materials

Novel loci and pathways significantly associated with longevity

Yi Zeng[1,2,# *], Chao Nie[3,#], Junxia Min[4,#],Xiaomin Liu[3,#], Mengmeng Li[5], Huashuai Chen[1,6], Hanshi Xu[3], Mingbang Wang[3], Ting Ni[7], Yang Li[8], Han Yan[8], Jin-Pei Zhang[8], Chun Song[8], Li-Qing Chi[8], Han-Ming Wang[8], Jie Dong[8], Gu-Yan Zheng[8], Li Lin[5], Feng Qian[5], Yanwei Qi[3,9], Xiao Liu[3], Hongzhi Cao[3], Yinghao Wang[3], Lijuan Zhang[3], Zhaochun Li[3], Yufeng Zhou[3], Yan Wang[3], Jiehua Lu[10], Jianxin Li[10], Ming Qi[4], Lars Bolund[3,11], Anatoliy Yashin[12], Kenneth C.Land[12], Simon Gregory[13], Ze Yang[14], William Gottschalk[15], Wei Tao[16], Jian Wang[3,17], Jun Wang[3,18], Xun Xu[3], Harold Bae[19], Marianne Nygaard[20],Lene Christiansen[20], Kaare Christensen[20], Claudio Franceschi[21], Michael W. Lutz[15], Jun Gu[16], Qihua Tan[20], Thomas Perls[22], Paola Sebastiani[23], Joris Deelen[24],Eline Slagboom[24], Elizabeth Hauser[13], Huji Xu[5], Xiao-Li Tian[8 *], Huanming Yang[3,17 *], James W. Vaupel[25]

[1] Center for the Study of Aging and Human Development, Medical School of Duke University, Durham, North Carolina, USA.[2] Center for Healthy Aging and Development Studies, National School of Development, Peking University, Beijing, China. [3] BGI-Shenzhen, Shenzhen, China. [4] The First Affiliated Hospital, Institute of Translational Medicine, School of Medicine, Zhejiang University, Hangzhou, China.[5] Department of Rheumatology and Immunology, Shanghai Changzheng Hospital, The Second Military Medical University, Shanghai, China. [6] Business School of Xiangtan University, Xiangtan, China. [7] State Key Laboratory of Genetics Engineering & MOE Key Laboratory of Contemporary Anthropology, School of Life Sciences, Fudan University, Shanghai, China. [8] Department of Human Population Genetics, Institute of Molecular Medicine, Peking University, Beijing, China. [9] Department of Bio-Medical Engineering, School of Life Sciences, Anhui Medical University, Hefei, China.[10] Department of Sociology, Peking University, Beijing, China. [11]Department of Biomedicine, Aarhus University, Aarhus, Denmark.[12]Population Research Institute, Duke University, Durham, North Carolina, USA.[13] Duke Molecular Physiology Institute, Medical Center, Duke University, Durham, North Carolina, USA. [14] National Institutes of Geriatrics, Beijing Hospital, Ministry of Health, Beijing, China.[15] Department of Neurology, Medical Center, Duke University, Durham, North Carolina, USA. [16]School of Life Sciences, Peking University, Beijing, China.[17] James D. Watson Institute of Genome Sciences, Hangzhou, China [18]Department of Biology, University of Copenhagen, Copenhagen, Denmark.[19] College of Public Health and Human Sciences, Oregon State University,[20] The Danish Aging Research Centre，

Unit of Epidemiology, Biostatistics and Biodemography, Institute of Public Health, University of Southern Denmark, Odense, Denmark. [21]Department of Experimental, Diagnostic and Specialty Medicine and Interdepartmental Centre 'L. Galvani', University of Bologna, Bologna, Italy.[22] Geriatrics Section, Department of Medicine, Boston University School of Medicine, Boston, MA.[23] Department of Biostatistics, Boston University School of Public Health, Boston, MA.[24] Department of Molecular Epidemiology and Netherlands Consortium for Healthy Ageing, Leiden University Medical Center, Leiden, The Netherlands. [25] Max Planck Institute for Demographic Research, Rostock, Germany.
[#] indicates the authors who contributed equally to this work. * indicates the authors who jointly supervised this work. Correspondence should be addressed to Y.Z.(zengyi@duke.edu), H.Y.(yanghm@genomics.org.cn) or X.T.(tianxiaoli@pku.edu.cn).
The findings of this manuscript (including the SNPs and four major pathways significantly associated with longevity) were presented at the annual conference of Population Association of Americas, April 29 – May 2, 2015, San Diego, U.S.A. (Zeng et al. 2015).

Supplementary Table 1. Basic characteristics of gender and age structures of the CLHLS samples.

	Southern region of China						Northern region of China						Combined					
	Centenarians			Controls			Centenarians			Controls			Centenarians			Controls		
	M	F	Total	M	F	Total	M	F	Total	M	F	Total	M	F	Total	M	F	Total
n	278	785	1063	265	622	887	286	829	1115	508	904	1412	564	1614	2178	773	1526	2299
Mean age	101.2	102.9	102.5	50.4	50.0	50.1	101.8	103.2	102.9	47.9	47.1	47.3	101.5	103.1	102.7	48.7	48.2	48.4
SD	3.70	3.37	3.54	6.64	8.05	7.65	3.24	3.44	3.44	6.54	7.38	7.10	3.48	3.41	3.49	6.68	7.79	7.44

Notes: M--Males; F--Females; n--Sub-sample size; SD--Standard deviation.

Supplementary Table 2. Gender-specific analysis for the 11 SNPs that are significantly associated with longevity and replicated in the Southern and Northern datasets.

SNP	Chr.	Nearby gene	Males			Females			p value of gender difference
			MAF(case /control)	*P*	Odds ratio	MAF(case /control)	*P*	Odds ratio	
rs2069837	7	*IL6* (intronic)	0.052/0.118	1.45E-06	0.473	0.053/0.084	2.02E-04	0.695	0.036
rs2440012	13	*ANKRD20A9P* (nc_exonic)	0.048/0.084	6.92E-04	0.547	0.056/0.084	1.30E-05	0.622	0.418
rs145672791	21	*MIR3156-3* (28 kb ownstream)	0.006/0.022	4.96E-03	0.282	0.003/0.016	9.48E-06	0.197	0.506
rs61856137	10	*AKR1C2* (27 kb upstream)	0.032/0.058	8.94E-03	0.569	0.029/0.055	6.56E-06	0.535	0.855
rs2704588	4	*FAM13A* (intronic)	0.001/0.020	3.68E-03	0.052	0.005/0.017	1.39E-04	0.331	0.066
rs1487614	4	*BEND4*(114 kb upstream)	0.112/0.142	2.63E-02	0.769	0.106/0.144	3.71E-06	0.696	0.594
rs10934524	3	*EPHA6* (383 kb upstream)	0.463/0.420	1.81E-02	1.227	0.462/0.409	8.48E-06	1.286	0.662
rs57681851	4	*ZFYVE28* (intronic)	0.174/0.136	9.66E-03	1.352	0.169/0.129	4.74E-05	1.354	0.849
rs7213812	17	*ASIC2* (intronic)	0.210/0.163	5.35E-03	1.325	0.191/0.152	1.71E-04	1.293	0.776
rs9568833	13	*OLFM4* (200 kb downstream)	0.162/0.192	4.13E-02	0.812	0.138/0.170	3.36E-04	0.773	0.755
rs405509	19	*APOE* (200 bp upstream)	0.342/0.295	5.49E-02	1.178	0.340/0.292	2.52E-04	1.224	0.957

Note: The *P* values of gender differences were derived by Woolf's test based on the gender-specific odds ratios (Methods/section M7).MAF; minor allele frequency.

Supplementary Table 3. Comparing the 11 independent loci associated with longevity in Han Chinese CLHLS GWAS datasets (Table 1) to the longevity GWAS datasets from white populations of European Union consortium and/or New England.

SNP	Chr	position	Nearest gene	Coded/ noncoded Allele	**Han Chinese Combined**			**EU longevity GWAS**		**New England GWAS**	
					MAF (case/ control)	*P*	Odds ratio	Direction of effect allele	*P*	Odds ratio	*P*
rs2069837	7	22768027	*IL6* (intronic)	G/A	0.053/0.095	1.80E-09	0.610	+	0.90	0.88	0.25
rs2440012	13	19440123	*ANKRD20A9P* (nc_exonic)	G/C	0.054/0.084	3.73E-08	0.602	NA	NA	NA	NA
rs145672791	21	14750023	*MIR3156-3* (28 kb downstream)	A/G	0.004/0.018	8.95E-08	0.219	NA	NA	0.39	0.25
rs61856137	10	5087978	*AKR1C2* (27 kb upstream)	T/G	0.029/0.056	1.60E-07	0.544	NA	NA	NA	NA
rs2704588	4	89849772	*FAM13A* (intronic)	C/T	0.004/0.018	2.38E-07	0.248	NA	NA	NA	NA
rs1487614	4	42269480	*BEND4* (114 kb upstream)	T/C	0.107/0.143	2.87E-07	0.716	-	0.066	0.93	0.23
rs10934524	3	96150160	*EPHA6* (383 kb upstream)	T/C	0.462/0.413	5.33E-07	1.266	+	0.98	1.01	0.46
rs57681851	4	2290698	*ZFYVE28* (intronic)	G/T	0.170/0.131	1.83E-06	1.348	NA	NA	NA	NA
rs7213812	17	31448649	*ASIC2* (intronic)	C/A	0.196/0.155	6.33E-06	1.29	+	0.275	NA	NA
rs9568833	13	53827016	*OLFM4* (200 kb downstream)	T/C	0.144/0.177	1.77E-05	0.778	NA	NA	0.83	0.46
**rs405509**	**19**	**45408836**	***APOE* (200 bp upstream)**	**G/T**	**0.341/0.293**	**3.64E-05**	**1.210**	**+**	**2.75E-06**	**1.29**	**2.46E-03**

Notes: (1)In the EU longevity GWAS, "+" means the allele is more frequent in individuals >=90 years of age as compared to individuals <65 years of age, while "-" means the opposite. (2) NA; not available, MAF; minor allele frequency.(3) rs1487615 and rs11652197 were used as proxy SNPs for rs1487614 and rs7213812 with $r^2 = 1$ and $r^2 = 0.97$, respectively, in the columns of EU longevity GWAS (according to the database, http://snipa.helmholtz-muenchen.de/snipa/index.php?task=proxy_search), since the SNPs of rs1487614 and rs7213812 were not available in the EU longevity GWAS. For the other SNPs which were not available (marked as "NA") in the EU longevity GWAS and/or New England GWAS, we did not find the proxy SNPs with $r^2>0.8$ using the database.

Supplementary Table 4. The independent SNPs associated with longevity ($P<10^{-4}$) identified in the Han Chinese CLHLS GWAS Southern-Northern combined dataset with p< 0.05 in the EU or New England longevity GWAS.

SNP	Chr	position	Nearby gene	Effect Allele	Not Effect Allele	**Han Chinese**			**EU GWAS**		**New England GWAS**	
						MAF (case/ control)	*P*	Odds ratio	Dir. effect allele	*P*	Odds ratio	*P*
rs4420638	19	45422946	*APOC1*	G	A	0.086/0.112	7.85E-05	0.746	-	4.09E-21	0.326	1.03E-09
rs9989350	15	97589967	*SPATA8,LOC91948*	G	C	0.235/0.277	6.09E-05	0.820	-	0.006	NA	NA
rs12650823	4	42253022	*BEND4,SHISA3*	T	A	0.094/0.122	6.09E-05	0.755	-	0.010	NA	NA
rs3114020	4	89083666	*ABCG2*	T	C	0.377/0.330	5.93E-05	1.195	+	0.022	NA	NA
rs4696616	4	155801039	*RBM46,NPY2R*	A	T	0.132/0.163	9.86E-05	0.785	-	0.041	NA	NA
rs931612	11	91832242	*MIR4490,FAT3*	T	C	0.097/0.073	6.13E-05	1.363	+	0.018	NA	NA
rs12739724	1	5038051	*AJAP1,MIR4417*	A	G	0.062/0.086	1.56E-05	0.695	-	0.018	1.19	0.187

Notes: the same as in Supplementary Table 3.

Supplementary Table 5. The 14 SNPs in theTOMM40/APOE/APOC1 region associated with longevity (p< 10^{-4}) identified in the CLHLS GWAS combined dataset, which are either previously reported longevity SNPs or are in very high linkage disequilibrium with the longevity SNPs reported in the EU and New England GWAS.

Chrom. location	SNP	CLHLS GWAS		EU GWAS		NECS GWAS		References
		P	OR	*P*	OR	*P*	OR	
19q13	rs4420638	7.85 x 10^{-5}	0.74	3.40 × 10^{-36}	0.72	1.03 x 10^{-9}	0.33	[1], [2]
19q13	rs2075650	1.74 x 10^{-5}	0.71	4.46 x 10^{-14}	<1.0	4.92 x 10^{-8}	0.44	[1], [2]
19q13	rs6857	3.28 x 10^{-5}	0.71	5.11 x 10^{-17}	<1.0	NA	NA	[1]
19q13	rs405509	3.64 x 10^{-5}	1.21	8.51 x 10^{-5}	>1.0	2.46 x 10^{-3}	1.29	[1], [2]

Chrom. location	SNPs newly identified in CLHLS GWAS			in very high linkage disequilibrium with longevity SNPs reported in EU and New England GWAS		References
	SNP	*P*	OR	Reported SNPs	r^2	
19q13	rs73052335	1.23 x 10^{-5}	0.71	rs4420638	0.99	[1], [2]
19q13	rs71352238	1.66 x 10^{-5}	0.71	rs6857	1.0	[1]
19q13	rs34404554	2.39 x 10^{-5}	0.71	rs2075650	1.0	[1], [2]
19q13	rs10414043	3.27 x 10^{-5}	0.72	NA	NA	
19q13	rs7256200	4.04 x 10^{-5}	0.72	rs405509	1.0	[1], [2]
19q13	rs12721046	4.18 x 10^{-5}	0.73	rs4420638	1.0	[1], [2]
19q13	rs7259620	4.44 x 10^{-5}	1.21	rs405509	0.9	[1], [2]
19q13	rs11556505	4.96 x 10^{-5}	0.72	rs6857	0.99	[1]
19q13	rs56131196	5.93 x 10^{-5}	0.74	rs4420638	1.0	[1], [2]
19q13	rs12721051	6.45 x 10^{-5}	0.75	NA	NA	

Note: For these 14 SNPs in the TOMM40-APOE-APOC1 region that were significantly associated with longevity in both our GWAS and previously reported studies, we conducted a haplotype analysis in which the haplotype blocks were defined on the basis of the method of reference [3] and visualized using Haploview [4].

References:

[1].Deelen, J. *et al*. Genome-wide association meta-analysis of human longevity identifies a novel locus conferring survival beyond 90 years of age. *Hum. Mol. Genet*. **23**, 4420-32 (2014).

[2].Sebastiani, P.*et al*. Genetic signatures of exceptional longevity in humans. *PLoS One***7**, e29848 (2012).

[3] Gabriel, S.B. *et al.* The structure of haplotype blocks in the human genome. *Science* **296**, 2225–2229 (2002).

[4] Barrett, J.C. *et al*. Haploview: analysis and visualization of LD and haplotype maps. *Bioinformatics***21**, 263–265 (2005).

Supplementary Table 6. The 25 canonical pathways enriched in longevity traits.

	Pathway/Gene Set Name	Description	*P*-value	FDR	Significant genes /Selected genes /All genes
Starch, Sucrose and xenobiotic metabolism	STARCH_AND_SUCROSE_ METABOLISM	UGT1A8,UGT1A10,UGT1A7,UGT1A9,UGT1A1, UGT1A6,UGT1A3,UGT1A5,UGT1A4,ENPP1	<0.001	0	10/23/44
	HSA00500_STARCH_AND_ SUCROSE_METABOLISM	UGT1A8,UGT1A10,UGT1A7,UGT1A9,UGT1A6, UGT1A3,UGT1A5,UGT1A4,UGT1A1,UGT2A1,ENPP1	<0.001	0.001	11/35/84
	HSA00040_PENTOSE_AND_ GLUCURONATE_INTERCONVERSIONS	UGT1A8,UGT1A10,UGT1A7,UGT1A9,UGT1A1, UGT1A6,UGT1A3,UGT1A5,UGT1A4,UGT2A1	<0.001	0	10/13/25
	IRINOTECAN_PATHWAY_ PHARMGKB	ABCG2,UGT1A8,UGT1A10,UGT1A7,UGT1A9, UGT1A6,UGT1A3,UGT1A5,UGT1A4,UGT1A1	<0.001	0	10/12/18
	HSA00980_METABOLISM_OF_ XENOBIOTICS_BY_CYTOCHROME_P450	AKR1C3,UGT1A8,UGT1A10,UGT1A7,UGT1A9,UGT1A6, UGT1A3,UGT1A5,UGT1A4,UGT1A1,UGT2A1,ADH4	<0.001	0	13/21/41
	HSA00860_PORPHYRIN_AND_ CHLOROPHYLL_METABOLISM	UGT1A8,UGT1A10,UGT1A7,UGT1A9,ALAD,UGT1A1, UGT1A6,UGT1A3,UGT1A5,UGT1A4,,UGT2A1,FECH,EPRS	<0.001	0	13/21/41
	PORPHYRIN_AND_CHLOROPHYLL _METABOLISM	UGT1A8,UGT1A10,UGT1A7,UGT1A9,ALAD,UGT1A1, UGT1A6,UGT1A3,UGT1A5,UGT1A4,FECH,EPRS	< 0.001	0	12/14/26
	HSA00604_GLYCOSPHINGOLIPID_ BIOSYNTHESIS_GANGLIOSERIES	ST6GALNAC4,GLB1,ST6GALNAC3,ST6GALNAC6	<0.001	0.001	4/11/16
	HSA00150_ANDROGEN_AND_ ESTROGEN_METABOLISM	UGT1A8,UGT1A10,UGT1A7,UGT1A9,UGT1A6, UGT1A3,UGT1A5,UGT1A4,UGT1A1,UGT2A1	0.001	0.018	10/31/54
	HSA00760_NICOTINATE_AND_ NICOTINAMIDE_METABOLISM	BST1,NMNAT2,ENPP1,NT5C2	0.001	0.0177	4/12/24
Immune response and inflammation	STATIN_PATHWAY_PHARMGKB	APOC1,APOE,APOA4,APOC3	< 0.001	0.0001	4/9/20
	HSA04940_TYPE_I_DIABETES_ MELLITUS	HLA-DRA,HLA-DPB1,HLA-B,HLA-A,HLA-F,GZMB, HLA-G,HLA-DRB1,CD28,HLA-DQB1,HLA-C,GAD2	< 0.001	0.0006	12/29/45
	HSA04514_CELL_ADHESION_ MOLECULES	MPZ,PVRL2,CNTN1,HLA-DRA,HLA-DPB1,HLA-B,HLA-A,CNTNAP2,CDH4, HLA-F,MAG,CLDN20,HLA-G,ITGA6,HLA-DRB1,GLG1,CLDN15,CD28, ITGB2,NRXN1,HLA-DQB1,CDH15,HLA-C,CLDN14,NEO1	<0.001	0.001	25/79/134
	ERYTHPATHWAY	IL6,TGFB2	<0.001	0.001	2/10/15
	HSA04612_ANTIGEN_PROCESSING _AND_PRESENTATION	HLA-DRA,HLA-DPB1,HLA-B,HLA-A,HLA-F, CANX,HLA-G,HLA-DRB1,HLA-DQB1,HLA-C	<0.001	0.001	10/34/83
	INFLAMPATHWAY	IL6,HLA-DRA,TGFB2,HLA-DRB1	0.004	0.0240	4/16/29
	HSA04060_CYTOKINE_CYTOKINE _RECEPTOR_INTERACTION	IL6,CXCL13,TNFRSF10C,BMPR1A,TNFRSF11B,IL2RB,VEGFA,GHR, TNFRSF1B,TNFSF13B,IL15RA, IL1RAP,GF, TNFRSF21,PLEKHO2,TGFB2, BMPR1B, IL28RA	0.001	0.0259	18/114/257
MAPK	P38MAPKPATHWAY	MAP3K1,MAP3K7,TGFB2,TRAF2,MEF2B	0.003	0.0357	5/22/40
	IL1RPATHWAY	IL6,MAP3K1,MAP3K7,TGFB2,IL1RAP	<0.001	0.0173	5/17/33
	ST_TUMOR_NECROSIS_FACTOR_ PATHWAY	TNFRSF1B,MAP3K7,TRAF2	<0.001	0.0110	3/12/29
	APOPTOSIS	MAP3K1,BIRC5,TNFRSF1B,GZMB,TNFRSF21,IRF2,IRF1	<0.001	0.0113	7/26/71
Calcium signaling	NO1PATHWAY	PDE3A,CALM3,RYR2,PRKG2,BDKRB2,PRKG1	< 0.001	0.0009	6/15/31
	CACAMPATHWAY	CALM3,CAMKK1,CAMK2A	0.006	0.0448	3/10/14
The other 2 enriched pathways	MRNA_PROCESSING_REACTOME	CLK4,DHX16,METTL3,SNRPN,SNURF,BRUNOL4,LSM2,CUGBP2	0.004	0.0273	8/37/124
	PROSTAGLANDIN_AND_ LEUKOTRIENE_METABOLISM	AKR1C3,LTA4H,PLA2G5	<0.003	0.0074	3/13/32

Supplementary Table 7. The 35 highly-represented genes (containing 531 SNPs associated with longevity with P<0.01) among the 25 canonical pathways enriched in longevity traits.

Occurrences in enriched pathways	Gene ID	Gene name
8	UGT1A10	UDP glucuronosyltransferase 1 family, polypeptide A10
8	UGT1A1	UDP glucuronosyltransferase 1 family, polypeptide A1
8	UGT1A3	UDP glucuronosyltransferase 1 family, polypeptide A3
8	UGT1A4	UDP glucuronosyltransferase 1 family, polypeptide A4
8	UGT1A5	UDP glucuronosyltransferase 1 family, polypeptide A5
8	UGT1A6	UDP glucuronosyltransferase 1 family, polypeptide A6
8	UGT1A7	UDP glucuronosyltransferase 1 family, polypeptide A7
8	UGT1A8	UDP glucuronosyltransferase 1 family, polypeptide A8
8	UGT1A9	UDP glucuronosyltransferase 1 family, polypeptide A9
5	UGT2A1	UDP glucuronosyltransferase 2 family, polypeptide A1
5	TGFB2	transforming growth factor, beta 2
4	IL6	interleukin 6
4	HLA-DRA	major histocompatibility complex, class II, DR alpha
4	HLA-DRB1	major histocompatibility complex, class II, DR beta 1
3	HLA-A	major histocompatibility complex, class I, A
3	HLA-B	major histocompatibility complex, class I, B
3	HLA-C	major histocompatibility complex, class I, C
3	HLA-DPB1	major histocompatibility complex, class II, DP beta 1
3	HLA-DQB1	major histocompatibility complex, class II, DQ beta 1
3	HLA-F	major histocompatibility complex, class I, F
3	HLA-G	major histocompatibility complex, class I, G
3	ENPP1	Ectonucleotide pyrophosphatase/phosphodiesterase 1
3	TNFRSF1B	tumor necrosis factor receptor superfamily, member 1B
3	MAP3K1	mitogen-activated protein kinase kinasekinase1
3	MAP3K7	mitogen-activated protein kinase kinasekinase 7
2	ALAD	aminolevulinate, delta-, dehydratase
2	CD28	CD28 molecule
2	EPRS	glutamyl-prolyl-tRNAsynthetase
2	FECH	ferrochelatase (protoporphyria)
2	AKR1C3	aldo-keto reductase family 1, member C3
2	CALM3	calmodulin 3
2	IL1RAP	interleukin 1 receptor accessory protein
2	GZMB	granzyme B
2	TNFRSF21	tumor necrosis factor receptor superfamily, member 21
2	TRAF2	TNF receptor-associated factor 2

Supplementary Table 8. eQTL results in ASIC2 loci.

GENE	SNPRS	OR	P value in our study	SNP Chr.	SNP Position	Probe GI	Probe Chr.	Probe Position	Gene	*P*-value	R-squared
ASIC2	rs7224279	1.201	1.4E-04	17	31450714	ILMN_1747078	11	1.26E+08	HYLS1	3.88E-09	0.2273
	rs11658301	1.204	1.2E-03	17	31450188	ILMN_1747078	11	1.26E+08	HYLS1	3.88E-09	0.2273

Supplementary Table 9. Potential eQTLs among the significant SNPs ($P<10^{-3}$) associated with longevity traits in 35 high-represent genes for pathways by querying the Genotype-Tissue Expression (GTEx) database derived from multiple cells and tissue types (lymphoblastoid cell lines, brain tissue and human fibroblasts) (The Genotype-Tissue Expression Consortium, 2013).

Gene	SNPRS	OR	P value in our study	SNP Chr.	SNP Position	Probe GI	Probe Chr.	Probe Position	*P*-value in eQTL	R-squared
MAP3K1	rs10055224	1.213	2.55E-04	5	56081200	ILMN_1757636	5	56205099	3.90E-08	0.1998
	rs10940513	1.215	2.31E-04	5	56082414	ILMN_1757636	5	56205099	3.90E-08	0.1998
	rs2408597	1.212	2.18E-04	5	56098631	ILMN_1757636	5	56205099	3.55E-08	0.2009
	rs3309	1.207	3.06E-04	5	56092778	ILMN_1757636	5	56205099	3.90E-08	0.1998
	rs4146565	1.212	2.25E-04	5	56093824	ILMN_1757636	5	56205099	3.90E-08	0.1998
	rs6870175	1.209	2.78E-04	5	56093370	ILMN_1757636	5	56205099	3.90E-08	0.1998
	rs6888317	1.209	2.78E-04	5	56093306	ILMN_1757636	5	56205099	3.90E-08	0.1998
	rs6893754	1.211	2.30E-04	5	56100005	ILMN_1757636	5	56205099	3.55E-08	0.2009
	rs96844	1.188	6.22E-04	5	56196603	ILMN_1757636	5	56205099	3.02E-10	0.2537

Reference: Genotype-Tissue Expression Consortium: The Genotype-Tissue Expression (GTEx) project. *Nat Genet*. **45**, 580–585 (2013).

Supplementary Table 10. Potential eQTLs among the significant SNPs ($P<10^{-3}$) associated with longevity traits in 35 highly-represented genes for pathways by querying the seeQTL database (Sullivan and Wright, 2012).

Gene	SNP	OR	P value in our study	chr	bp_SNP	Entrez Gene ID	Gene Symbol	Qvalue
HLA-B	rs2844586	0.7429	1.65E-04	6	31318023	54535	CCHCR1	6.86E-02
HLA-DPB1	rs3117242	0.8529	7.95E-04	6	33069892	3115	HLA-DPB1	2.42E-10
MAP3K1	rs10940511	1.197	7.70E-04	5	56012879	133383	C5orf35	2.27E-03
MAP3K1	rs11960484	1.248	8.59E-04	5	56109153	133383	C5orf35	8.08E-05
MAP3K1	rs252905	1.251	7.63E-04	5	56118874	133383	C5orf35	6.58E-05
MAP3K1	rs252906	1.253	7.05E-04	5	56119189	133383	C5orf35	4.60E-04
MAP3K1	rs4700008	1.218	3.26E-04	5	56044185	133383	C5orf35	1.24E-02
MAP3K1	rs6862199	1.197	7.67E-04	5	56009011	133383	C5orf35	9.11E-04
MAP3K1	rs702689	1.225	1.00E-04	5	56177442	133383	C5orf35	9.00E-06
MAP3K1	rs702691	1.251	7.63E-04	5	56114525	133383	C5orf35	6.58E-05
MAP3K1	rs832552	1.253	7.32E-04	5	56113849	133383	C5orf35	3.74E-04
MAP3K1	rs832566	1.269	3.94E-04	5	56151743	133383	C5orf35	2.41E-04
MAP3K1	rs832577	1.268	4.05E-04	5	56163786	133383	C5orf35	2.89E-04
MAP3K1	rs832585	1.252	7.58E-04	5	56125903	133383	C5orf35	7.39E-04

Reference: Sullivan, P.F. & Wright FA. seeQTL: A searchable database for human eQTLs. Bioinformatics 28, 451-452 (2012).

Supplementary Table 11. Potential eQTLs among the significant SNPs ($P<10^{-3}$) associated with longevity traits in 35 high-represent genes for pathways by querying the eQTL database from the Gilad/Pritchard group at University of Chicago (http://eqtl.uchicago.edu/Home.html).

Pathway_Gene	SNP RS	OR	P value in our study	Chr.	study	SNP Position	Score
HLA-B	rs114283068	0.7439	2.28E-04	chr6	Veytieras_PVALUE_eqtl	31317024	10.3263716

Supplementary Figures

PC1 VS PC2

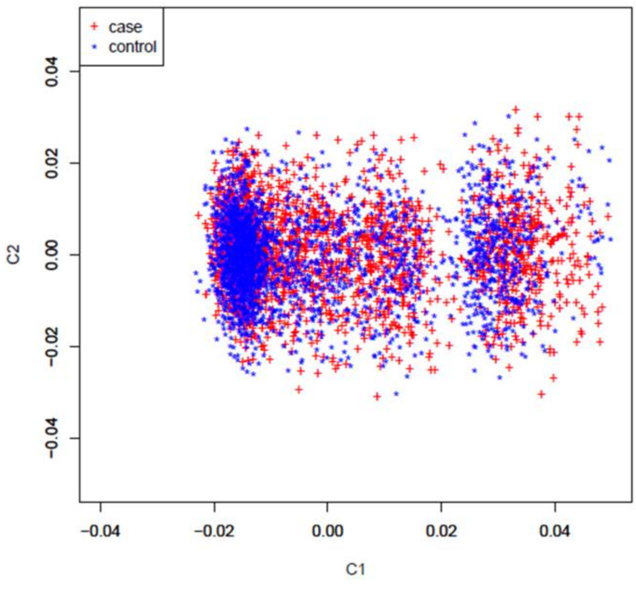

PC1 VS PC3

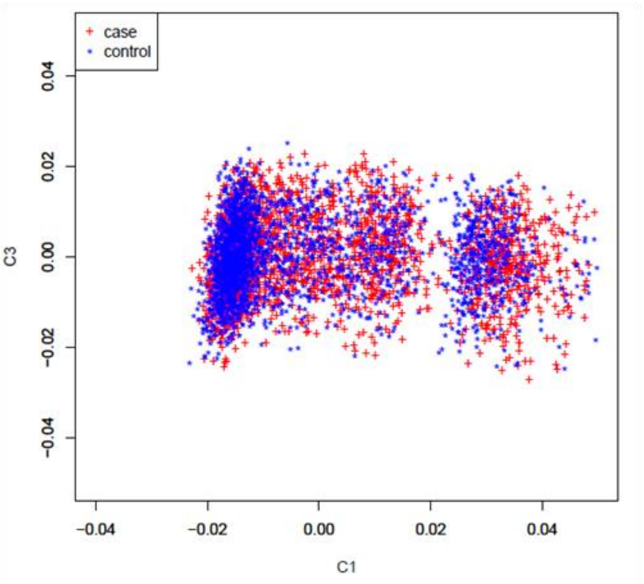

PC2 VS PC3

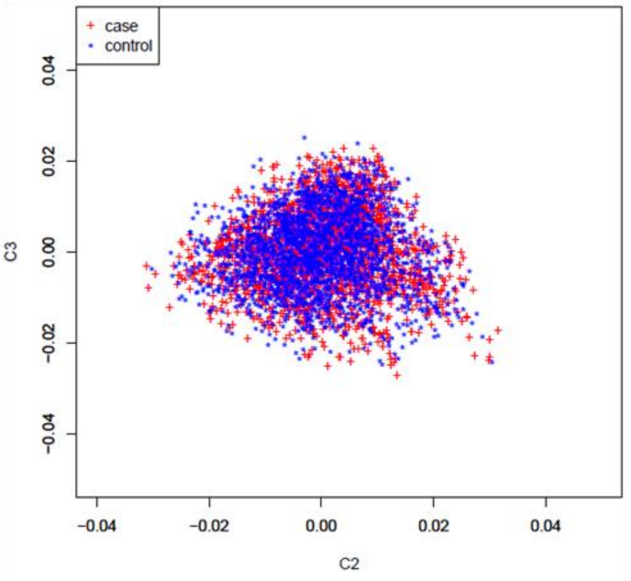

Supplementary Figure 1. Results of principal components analysis (PCA) in the case and control samples for the combined dataset. PCA was performed on the 4,477 GWAS samples (2,178 cases and 2,299 controls), with cases and controls evenly distributed in clusters of PC1 vs. PC2, PC1 vs. PC3 and PC2 vs. PC3.

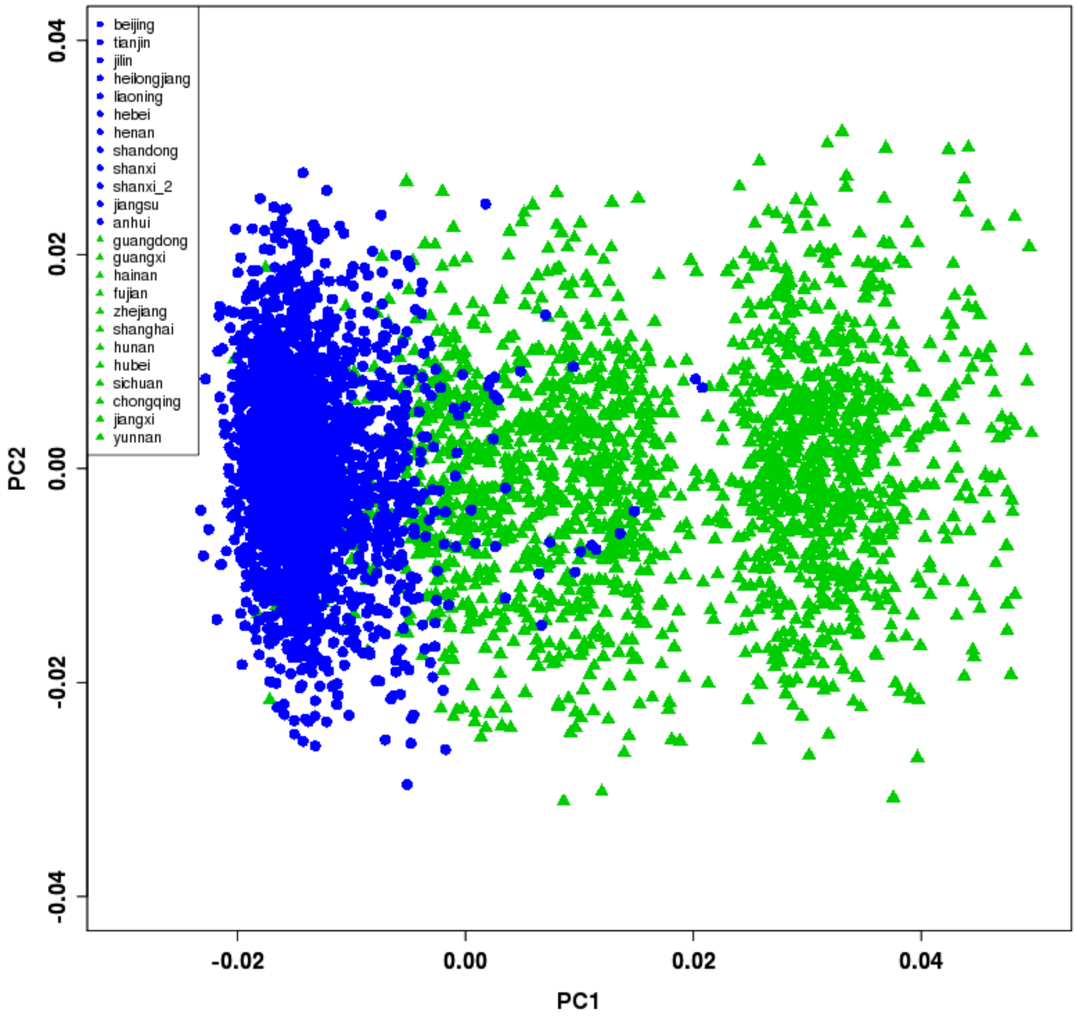

Supplementary Figure 2. Results of principle components analysis (PCA) of the 4,477 valid samples.

Note: Green color indicates “Southern region”; Blue color indicates “Northern region”.

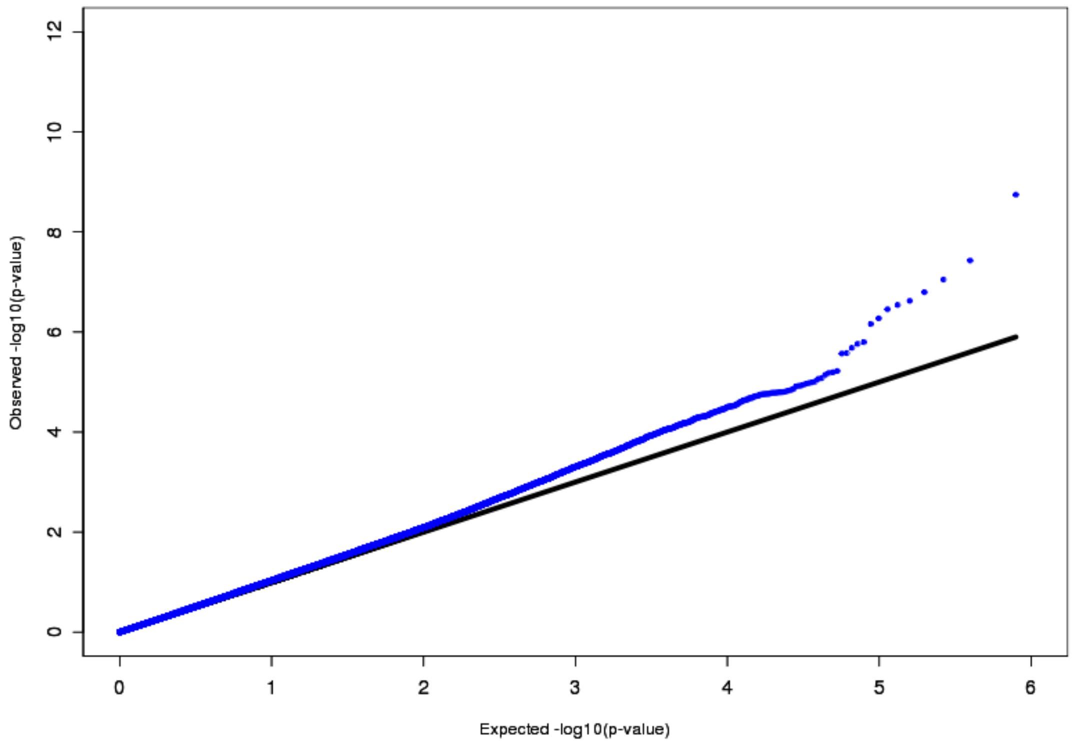

Supplementary Figure 3. Quantile-quantile plot with observed –log10 (p values) and the expected –log10 (p values) in the genotyped combined dataset. The genomic inflation factor is 1.022 (λ=1.022).

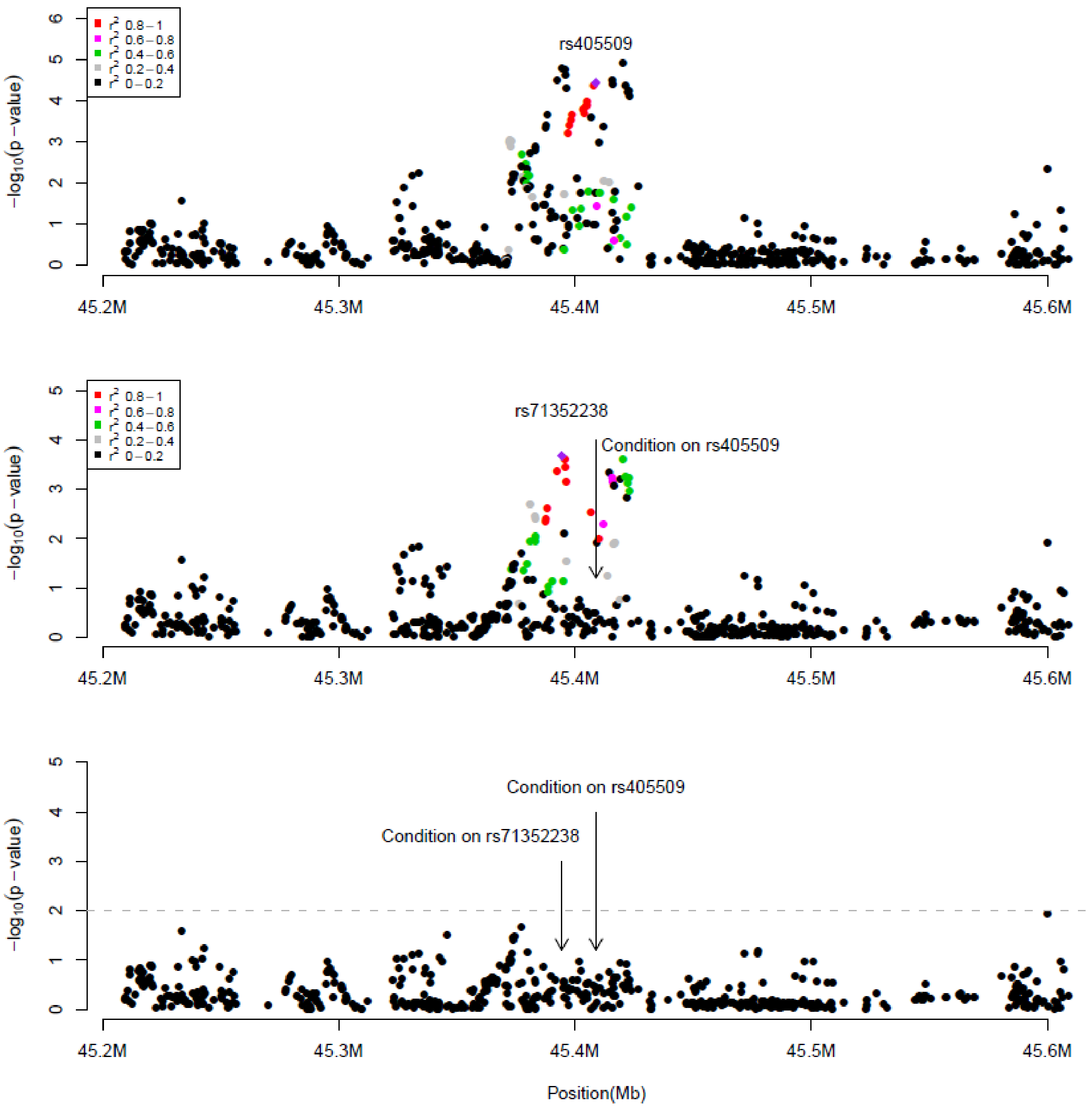

Supplementary Figure 4.Two independent signals in TOMM40- APOE-APOC1 region.
